# Integrative multi-omics analysis of growth plate regulation underlying body size in miniature pigs

**DOI:** 10.1038/s42003-026-10538-9

**Published:** 2026-06-30

**Authors:** Nadia Khaveh, Jan Berghöfer, Brehm Ralph, René Buschow, Karsten Cirksena, Cord Drögemüller, Abeera Fatima, Gordon Grabert, Gisa Gerold, Alexander Grahofer, Ralf Herwig, Klaus Jung, Tim Kacprowski, Bai-Wei Lo, Roya Shiasi Sardoabi, Martin Vingron, Stefan Mundlos, Julia Metzger

**Affiliations:** 1 Max Planck Institute for Molecular Genetics, Berlin, Germany; 2 Institute of Animal Genomics, University of Veterinary Medicine Hannover, Hannover, Germany; 3 Institute of Chemistry and Biochemistry, Department of Biology, Chemistry and Pharmacy, Freie Universität Berlin, Berlin, Germany; 4 Institute for Anatomy, University of Veterinary Medicine Hannover, Hannover, Germany; 5 Helmholtz Centre for Infection Research, Braunschweig, Germany; 6 Institute of Genetics, Vetsuisse Faculty, University of Bern, Bern, Switzerland; 7 Institute of Data Science in Biomedicine, TU Braunschweig, Braunschweig, Germany; 8 Braunschweig Integrated Centre for Systems Biology (BRICS), TU Braunschweig, Braunschweig, Germany; 9 Institute of Virology, Medical University of Innsbruck, Innsbruck, Austria; 10 Clinic for Swine, Department of Clinical Veterinary Science, Vetsuisse Faculty, University of Bern, Bern, Switzerland

**Keywords:** Gene expression profiling, Epigenetics, Data integration

## Abstract

Body size represents a complex phenotype driven by genetic variation and epigenetic regulation, with the molecular processes underlying this trait remaining a central challenge to disentangle. To elucidate these fundamental mechanisms, we apply a multi-omics approach that combines ROH-based selection mapping with growth plate epigenomics in pigs. Taking advantage of divergent selection that separates pigs into miniature and larger-sized groups, we target genomic regions under this intense selection for height, which harbour functional variants with pronounced effects. We assemble a multi-omics dataset, identifying homozygous alternative SNPs in Aachen Minipigs and Mini-LEWE predicted to affect cis-regulatory elements potentially interacting with differentially expressed genes that drive body size in breed-dependent ways. Our results point to an lncRNA (*ENSSSCG00000048200*) near *SDR16C5* and *PLAG1, HPX* and NET-related pathways, as central players in the growth plates. In summary, our study offers a multi-layered characterisation of regulatory mechanisms in the growth plates in the pig model.

## Abstract (web summary)

Integrative multi-omics profiling of growth plates reveals genetic regulatory mechanisms underlying body size variation and miniaturisation in pigs.

## Introduction

Body size is a fundamental trait in mammals that reflects and impacts physiology, energy metabolism, performance and adaptation^1–3^. Variation in growth and stature arises from complex interactions between genetic and regulatory factors, manifesting as highly polygenic in humans, with many small effects, yet often driven by a few genes with large effects in domestic animals such as dogs and pigs^4–6^. The majority of these causative variants are noncoding and affect body size by altering the regulation of gene expression or translation^7–9^. These variants, attributed to the complex growth process, are typically assigned to cis-regulatory elements, where groups of transcription factors bind together and locally regulate chromatin accessibility, thereby controlling gene activity^10,11^.

Yet, tracking down the specific variants is challenging. Domestic animals offer a major advantage for dissecting such complex traits: strong artificial selection has left footprints in their genomes represented by unique runs of homozygosity (ROHs)^12,13^. These contiguous homozygous segments arise from recent selection or shared ancestry and are therefore powerful indicators of genomic regions affected by breeding^14,15^. Integrating ROH regions (ROHRs) into trait mapping refines the resolution of genetic association studies by prioritising variants most likely shaped by directional selection, thereby supporting the identification of functional loci for height^16,17^. This implies that the complexity of multiple drivers for the growth of a body can be broken down in highly selected breeds, where intense selection pressure exposes the key contributors^18,19^.

In particular, pigs (*Sus scrofa domesticus*) represent a powerful model, as divergent selection has shaped distinct breed types ranging from miniature pigs to commercial larger-sized animals^20^. Miniature pig breeds are bred for a broad range of characteristics, yet all share one defining feature, their small body size^21^. This trait is typically reflected by an average live weight of 45-100 kg within the breeding stocks^21^. It makes miniature pigs more comparable in size to humans^22^. Hence, they represent an ideal model for biomedical research due to their similar anatomy and physiology as well as an approximately threefold greater genome sequence similarity to humans, relative to that observed between mouse and human^22,23^. Studies addressing selection signatures underlying their characteristic miniature body size revealed distinct biological pathways across different breeds, suggesting that the mechanisms regulating size may vary between miniature pigs^24,25^. In addition, an earlier hypothesis of pituitary dwarfism^26^ as the origin of miniature pig breeds was rejected, as neither signals of selection were detected near *IGF* genes, nor differences in serum IGF1 or IGF2 levels were observed, and no evidence for a diminished response to growth hormone secretion was found^20,27^. In fact, the key regions for bone elongation are the growth plates near the ends of the long bones, which harbour chondrocytes undergoing distinct but time-limited developmental stages organised into zones, allowing the transfer from cartilaginous tissue to bone^28^. Several factors and gene expression patterns have been linked to growth plate regulation, although the extrapolation of the data obtained in experimental animals, e.g., mice, to the human growth plate was reported to be difficult, emphasising the need for additional model systems such as pigs^28^. These findings highlight that body size is not explained by a single pathway but instead represents a complex phenotype, whose genetic and regulatory determinants are far from fully understood.

In this study, we conducted a comprehensive multi-omics analysis of body size in pigs, focusing on the functionally active growth plates of the long bones. We analysed genomic variants in ROHs as signatures of selection for miniature size in one litter of each of the two miniature pig breeds, Aachen Minipig (AM) and Mini-LEWE (ML), and assigned them to function by integrating chromatin accessibility (ATAC-seq), three-dimensional genome organisation (Hi-C), transcriptome profiling (RNA-seq) and protein-protein interaction networks in comparison to larger-sized pigs. As the two miniature pig breeds were developed in different breeding programmes and may therefore harbour distinct genetic variants underlying reduced body size, analyses were first conducted separately to identify breed-specific genomic signals before evaluating shared signatures across miniature pigs.

This integrative framework, which combines ROH-based selection mapping with growth plate epigenomics, offers a novel perspective for linking selection signatures to regulatory mechanisms. By linking growth plate biology with the genomic and regulatory landscape, we elucidate how selection shapes body size, offering broader perspectives on mammalian growth and adaptation.

## Results

### Body size divergence and growth plate architecture in miniature and larger-sized breeds

**Fig. 1:**
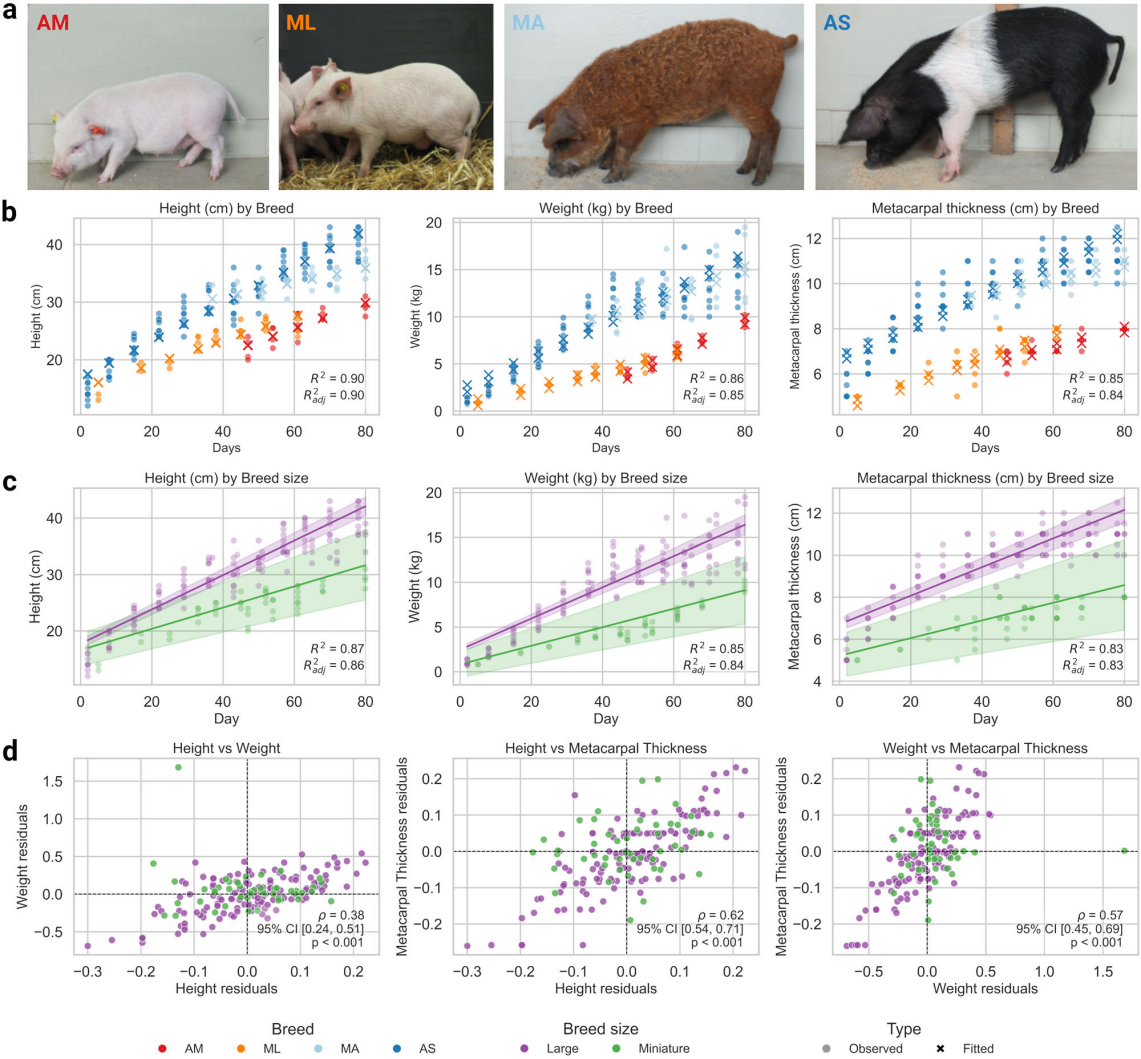
Growth trajectories and trait relationships in four pig breeds. **a** Piglets from all four breeds under study. The photos are downsized to reflect the actual size ratio among all four breeds. From left to right: Aachen Minipig (AM), Mini-LEWE (ML), Mangalitza (MA) and Angeln Saddleback (AS). **b** Development of height, weight, and metacarpal bone thickness over 80 days. Circles represent observed values and crosses represent fitted values from univariate linear mixed models. Model fit is summarised by the coefficient of determination (*R*^2^) and adjusted *R*^2^. **c** Results from a multivariate linear mixed model fitted jointly to all three traits. Breeds are grouped into large (AS, MA) and mini (AM, ML). Lines represent estimated fixed effects, with shaded areas showing 95% confidence intervals. **d** Correlations between growth traits were calculated from a multivariate model that accounts for breed size, sex and breed size × days interaction, and repeated measurements. Scatterplots represent correlations of Pearson residuals. n: AS = 9, MA = 4, AM = 4 and ML= variable (9 for weight and 4 for other parameters). *p*-values are noted on each plot when applicable.

We compared body size of two miniature pig breeds (Aachen Minipig, AM; Mini-LEWE, ML) with two larger-sized pig breeds (Mangalitza, MA; Angeln Saddleback, AS; Fig. 1a). One litter per breed was monitored, and piglets were measured every seven days from birth to 80 days of age for three morphological traits: body weight, height and metacarpal thickness (Supplementary Tables 1–12). A total of 26 piglets were recorded under identical housing and feeding conditions. After excluding incomplete records (where initial measurements could not be collected due to maternal behaviour constraints), the final dataset comprised 22 piglets with 180 repeated observations.

We first fitted univariate linear mixed models for each growth trait (height, weight, and metacarpal thickness) using days, sex, breed, and the breed × days interaction as covariates. Female sex and the AM breed were used as reference groups. The dependency structure was modelled with an independent and a compound symmetry component. All three traits showed significant positive effects of age (days), indicating continuous growth. Breed effects were also significant, particularly for the larger-sized breeds. Breed × days interactions were small but significant in some cases, suggesting subtle differences in growth trajectories across breeds. The coefficient of determination (adjusted *R²*) ranged from 0.84 to 0.90, indicating a good model fit across traits (Fig. 1b).

We then applied a multivariate linear mixed model to estimate trait correlations while simultaneously accounting for repeated measures, sex, and breed × days interactions, with breeds grouped by size (large: AS, MA; mini: AM, ML). The model confirmed the strong effects of days and breed and detected significant dispersion parameters, reflecting both individual heterogeneity and shared within-individual variation. The estimated correlation structure (Fig. 1c) revealed a moderate positive association between height and weight (*⍴* = 0.38, 95% CI [0.24, 0.51], *p*-value < 0.001). In contrast, correlations between metacarpal thickness and height (*⍴* = 0.62, 95% CI [0.54, 0.71], *p*-value < 0.001) and between metacarpal thickness and weight (*⍴* = 0.57, 95% CI [0.45, 0.69], *p*-value < 0.001) were more substantial (Fig. 1d). Since sufficient observations for the AS breed were available, we also fitted a multivariate model restricted to this breed to estimate the within-breed trait correlation structure. These correlations appeared stronger compared to the breed-adjusted estimates (height–weight *⍴* = 0.49, 95% CI [0.24, 0.51], *p*-value < 0.001; height–metacarpal thickness *⍴* = 0.72, 95% CI [0.63, 0.80], *p*-value < 0.001; weight–metacarpal thickness *⍴* = 0.72, 95% CI [0.59, 0.85]).

Taken together, the univariate analyses provided robust evidence for breed- and time-related effects on growth, while the multivariate approach uncovered additional dependencies between traits that could not be captured in separate models. Grouping breeds by size (Large: AS, MA; Miniature: AM, ML) further improved estimation by balancing data coverage across time points. Overall, the joint modelling strategy revealed both trait-specific components of growth trajectories and trait correlations in piglets.

**Fig. 2:**
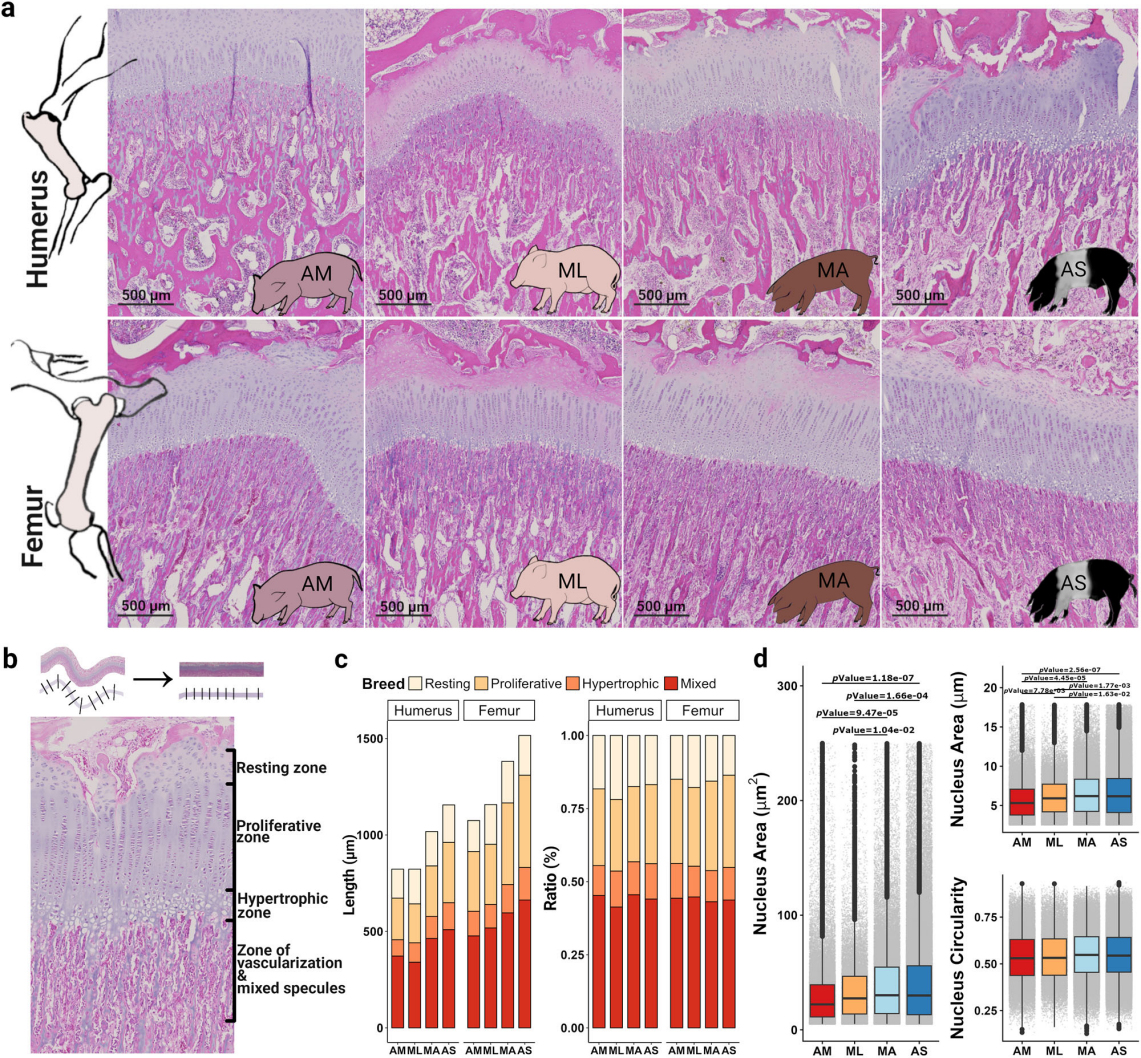
Growth plate histology among the four pig breeds under study. **a** Histological sections of the distal growth plates of the humerus and femur from all four breeds, stained with haematoxylin and eosin (H&E). **b** Schematic of the characteristic sigmoid shape of a growth plate and subsequent artificial reshaping of H&E-stained pictures in an accordion-like manner into a linear shape. Vertical alignment and unaltered pixel scaling were maintained to preserve the actual length and ratio of the investigated zones. **c** Length (µm) and relative length (ratio) of each distinct histological zone of the growth plates are shown with a bar plot based on bone type (humerus or femur) in each pig breed. **d** Means of nuclei area (µm^2^), diameter (µm) and circularity of measured nuclei of chondrocytes in the growth plates are shown in each pig breed, suggesting a larger nuclei area in larger-sized breeds. The central horizontal line of boxes represents the median. Individual observations are displayed as grey jittered points to illustrate the distribution of the raw data. Outlying observations are highlighted as black points. AS: *n* = 9, MA: *n* = 4, AM: *n* = 4 and ML: *n* = 9 for weight and *n* = 4 for other parameters. *p*-values are noted on each plot when applicable.

To further explore the biological basis of growth differences, we investigated the anatomical and histological features of the growth plates, the areas of longitudinal bone growth in the two long bones, the femur and humerus, across miniature and larger-sized breeds. All four breeds showed growth plates with four characteristically distinct zones: resting, proliferative, hypertrophic, and a zone of vascularisation (Fig. 2a, b). The length of each zone corresponded to the overall size of the pig breed. In contrast, the ratio of the zones to each other within individual growth plates remained consistent across animals of the same breed (Fig. 2c). Moreover, investigations of the nuclei areas and nuclei diameters of the chondrocytes within the cartilaginous regions of the growth plates revealed significantly smaller nuclei in the two miniature breeds (the smallest *p* < 0.03) compared to the two larger-sized breeds (Fig. 2d). AM showed a very small mean nuclear area of 30.24 µm², slightly but not significantly smaller than the mean nuclear area in ML (34.62 µm²). However, the mean nuclear diameter of chondrocytes from AM was considerably smaller (5.70 µm) in comparison to ML (6.16 µm). In the larger-sized pigs, we detected no significant difference in the nuclei areas or mean nuclei diameter between MA (mean nuclei area = 40.32 µm^2^, mean nuclei diameter = 6.57 µm) and AS (mean nuclei area = 42.17 µm^2^, mean nuclei diameter = 6.64 µm). In addition, the circularity of the nuclei from the chondrocytes was averaged to 0.5 in all breeds, suggesting no shrinkage or squishing occurred during tissue processing.

### Identification and prioritisation of candidate variants and target genes within runs of homozygosity regions

Short-read whole-genome sequencing (WGS) data from four AM and six ML as well as an additional 22 miniature pigs (from six breeds/populations), 65 large pigs (from 18 breeds), one MinipigXMangalitza crossbreed and 11 larger-sized crossbred pigs from all over the world were analysed (Supplementary Data 1). Quality metrics from short-read alignments revealed a mean alignment rate of 98.7% and consistent genome coverage across samples, with an average sequencing depth of 25.08X (minimum depth of 11.76X), providing sufficient coverage for accurate variant calling. In total, 52,042,820 SNPs and 12,250,578 INDELs were called from the dataset of 109 pigs. Additionally, we identified 304,204 structural variants (SVs) using Manta and 606,699 SVs using Smoove, both derived from the same sequencing data.

**Fig. 3:**
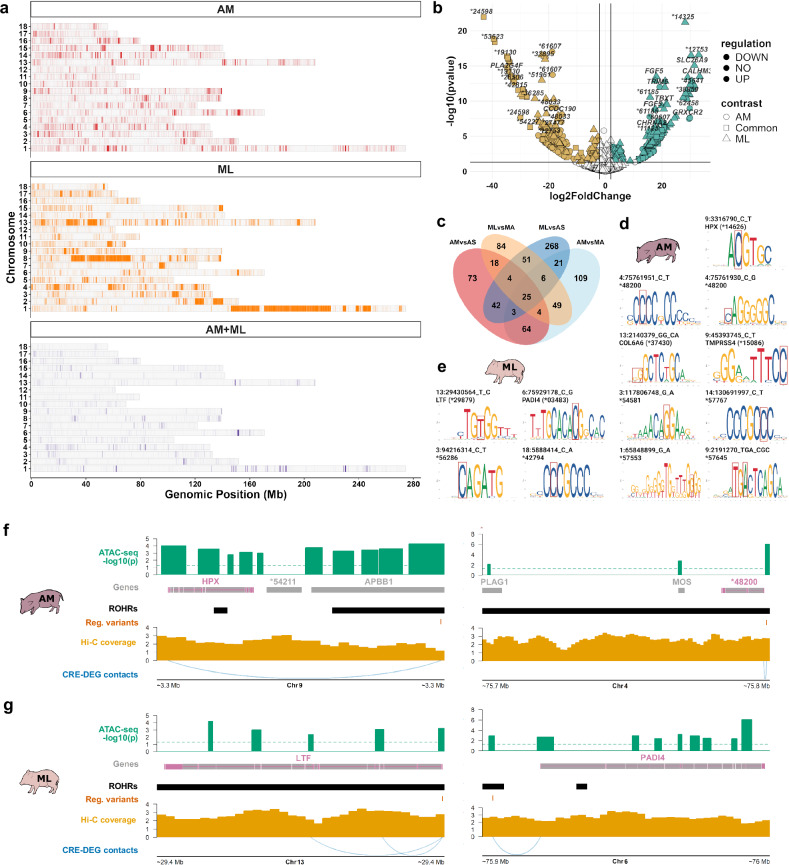
Multi-layered prioritisation strategy for the detection of key regulatory variants and target DEGs associated with miniaturisation. **a** Distribution of ROHRs for both miniature pig breeds individually (AM, ML) and shared by both (AM + ML). Grey bars represent all 18 pig autosomes with coloured segments indicating ROHRs. Only large ROHRs are visible in this graph. **b**, **c** DEGs in all four contrasts based on their log2FoldChange and *p*-value are shown in a volcano plot (significance threshold= |log2FoldChange | >2 and *p*-value < 0.05)., and the overlap of DEGs between each contrast is displayed in a Venn diagram. The label “common” in the volcano plot refers to genes which are significantly differentially expressed in all four contrasts in the innermost layer of the Venn diagram. **d**, **e** The most significant TF binding motif for each identified candidate variant in AM and ML, respectively. This section was created using BioRender. **f**, **g** Selected candidate gene loci with regulatory variants (hom. variants overlapping CRE indicated by OCR) within ROHRs assigned to their target DEGs with a gABC model using ATAC-Seq read counts and Hi-C contact frequencies (*=ENSSSCG000000). The green dashed line indicates the significance threshold for ATAC-Seq peaks (*p* < 0.05). n: refer to Supplementary Fig. 1.

To identify runs of homozygosity regions (ROHRs) in the miniature pigs of interest, a subset of 55 from 109 pig samples was selected (see Supplementary Data 1) meeting our criteria for a reliable SNP-set with sufficient variation for ROH calling. This SNP-set comprised 38,362,967 single-nucleotide polymorphisms (SNPs). Using our previously published simultaneous ROH detection approach^13^ with two tools, PLINK and RZooRoH, a total of 2,818,292 ROHs (parameter set PLINK_A), 759,039 ROHs (parameter set PLINK_B), and 2,820,518 ROHs (using RZooRoH) were called for the four AM samples (Fig. 3a). For the three ML samples, 3,723,765 ROHs (PLINK_A), 799,201 ROHs (PLINK_B), and 5,784,703 ROHs (RZooRoH) were identified. Subsequent ROHR analysis (Supplementary Data 2) identified 49,381 ROHRs in AM, accounting for 10.35% of the autosomal genome, and 50,669 ROHRs in ML, covering 19.69% of the autosomal genome. In the combined dataset of both miniature pig breeds of interest (AM + ML), 18,428 ROHRs were detected, accounting for 2.38% coverage of the autosomal genome.

In the next step, we checked these ROHR for overlap with variants of interest, taking into account all available genomic data from 109 pigs. Genotype filtering for homozygous alternative SNPs within ROHRs resulted in 20,892 SNPs and 3,294 INDELs for AM, 30,727 SNPs and 3955 INDELs for ML, and 615 and 88 INDELs for the combined AM + ML dataset (see also Supplementary Data 2). Among these, 22 SNPs in AM and 30 SNPs in ML were predicted to have coding effects (SIFT < 0.1, Supplementary Data 3). Furthermore, 35 SVs with homozygous alternative SNP genotypes overlapping with ROHRs in AM, 42 in ML, and two in the combined dataset AM + ML were identified using the variant calling results from Manta analysis. Among the variants called by Smoove within ROHR, a total of 307 SVs were found to be homozygous alternative SNPs in AM, 28 in ML, and 29 in AM + ML.

### Discovery and functional annotation of noncoding candidate variants

To elucidate the dynamics of gene regulation within the growth plate, we took biopsy punches of this tissue at distal humerus and femur from both miniature and larger-sized piglets. These samples underwent RNA-seq and Assay for Transposase-Accessible Chromatin sequencing (ATAC-seq) and were integrated as distinct layers of information to assign potential functions to the detected noncoding variants.

In total, 96 differentially expressed genes (DEGs) were called, including 57 lncRNAs, pseudo or novel genes passing the significance threshold in both contrasts AMvsAS and AMvsMA, of which eight DEGs passed the corrected FDR threshold (padj < 0.05). Additionally, 86 DEGs (68 were either lncRNA, pseudo- or novel genes) were identified in the contrasts MLvsAS and MLvsMA, of which 47 DEGs passed the corrected FDR threshold (Fig. 3b, c, Supplementary Data 4). Among these genes, 25 DEGs (including 13 lncRNAs and four novel genes) were common in all four contrasts.

In the next step, we intersected the subset of variants, which were homozygous alternative SNPs in the miniature pigs of interest and located within ROHR for these breeds, with ATAC‑Seq peaks indicating open chromatin regions (OCRs) and thus potential cis-regulatory elements (CREs), such as enhancers or promoters, and matched them to DEGs. Regulatory element-gene links were inferred using the generalised Activity-By-Contact (gABC) model, which integrates chromatin accessibility signals from ATAC-seq with chromatin contact frequencies derived from Hi-C (see Supplementary Data 5). In total, 23 variants detected in AM could be assigned to DEGs using this approach. These 23 variants were predicted to affect 11 CREs, which in turn showed a potential interaction with 11 DEGs in AM. Three of these 11 DEGs, Hemopexin (*HPX*)*, ENSSSCG00000048200* and *ENSSSCG00000057767*, were common in both contrasts AMvsAS and AMvsMA, and two lncRNAs (*ENSSSCG00000055080* and *ENSSSCG00000057553*) were identified as DEGs in all four contrasts (Supplementary Data 3). In the ML dataset, we identified 13 homozygous variants with a predicted effect on eight CREs assigned to seven genes designated as DEGs in at least one ML contrast (MLvsAS or MLvsMA). However, none of these variants appeared simultaneously in both MLvsAS and MLvsMA contrasts. Furthermore, no variants shared by both miniature pig breeds fulfilled the criteria of being located within a CRE and simultaneously assigned to a DEG.

Transcription factor (TF) footprinting highlighted 12 of the candidate variants in AM as potential causes of significantly altered TF binding affinities and subsequent significantly altered gene expression of a total of eight DEGs (Supplementary data 3, Fig. 3d, e). Among the candidate variants for ML, four variants were predicted to result in a modified binding activity of transcription factors, potentially affecting the gene expression of the DEGs, *Peptidyl Arginine Deiminase 4 (PADI4), Lactotransferrin (LTF), ENSSSCG00000056286* and *ENSSSCG00000042794*.

In addition, we found two homozygous 289 bp- and 92 bp-insertions in AM detected by Manta, which were located in intron 6 of the DEG *Paired Box gene-7* (*PAX7*, chromosome 6 at 77221746 bp) and intron 4 of the DEG *Phosphatase and Actin Regulator 1 (PHACTR1)* (chromosome 7 at 9367404 bp). Nevertheless, none of these genes were identified as DEG in AM, but exclusively in the comparison of MLvsMA, and were thereby not further pursued as candidates. No structural variants meeting the filtering criteria were identified using Smoove.

### Linking Fst-regions and GWAS-genes to noncoding variants

To address whether population differentiation due to genetic structure supports the identification of variants associated with height in miniature pigs, we also calculated genome-wide fixation indices (F_ST_) comparing miniature pig breeds jointly to larger-sized pigs (Supplementary Data 6).

In the first step, F_ST_ were calculated using the dataset from 109 pigs (excluding one unclassifiable hybrid pig) already used in the variant calling in this study. This analysis resulted in a total of 186 genomic windows with F_ST_ exceeding the significance threshold (top 0.2% of the empirical F_ST_ distribution). Intriguingly, we identified in the peak region on chromosome 4 at 75,490,001–82,570,000 bp the most significant SNP at 75,630,001-75,680,000 bp (weighted F_ST_: 0.7454), which directly overlapped with *Pleiomorphic Adenoma Gene 1 (PLAG1)* and *ENSSSCG00000032573*. Furthermore, the peak region also contained one of our highlighted noncoding candidate variants in AM (g.75761951:C > T, rs342683522) assigned to an enhancer element potentially modifying the expression of the long-noncoding RNA encoded by *ENSSSCG00000048200*, distal of *Oocyte Maturation Factor Mos (MOS)* and *PLAG1* and *Short Chain Dehydrogenase/Reductase Family 16 C Member 5 (SDR16C5)*.

It was interesting to find that *PLAG1* was also among the height-associated GWAS genes (GWAS catalogue) expressed in the growth plate, to which we could assign a homozygous alternative SNP (g.75646178:G > A, rs7605500220) in AM within ROHR. In this analysis of GWAS genes, we identified 65 homozygous alternative alleles that potentially affect 42 CREs, interacting with 33 expressed genes in AM linked to GWAS traits, hereafter referred as GWAS-expressed genes (Supplementary Data 7). Among these genes, we also identified *Aggrecan* (*ACAN*) and *T-Box Transcription Factor 5* (*TBX5*), which are assigned to CREs. For ML, 42 homozygous variants were identified, potentially affecting 32 CREs assigned to 28 GWAS-associated genes, including *Suppressor of Cytokine Signalling 2* (*SOCS2)*. In AM, 34 of these variants were suggested to result in significantly altered TF binding affinities (*p *< 0.05). Furthermore, 13 variants assigned to GWAS-expressed genes were also predicted to substantially affect TF binding affinities in ML. In addition, we detected eight homozygous SVs intersecting seven GWAS-expressed genes in AM and three homozygous SVs affecting two GWAS-expressed genes in ML.

In a second step, the F_ST_ analysis was extended to an expanded dataset comprising 201 pigs, including additional miniature pig populations and further larger-sized breeds for comparison, to evaluate the robustness of the observed differentiation signals in a broader population context. In this extended analysis, most of the differentiation peaks identified in the initial analysis were no longer detected above the significance threshold. However, the two peak regions with 349 F_ST_ window hits on chromosomes three and four remained significant, with slightly shifted genomic positions (chromosome 3: 38,710,001–43,790,000 bp; chromosome 4: 75,630,001–82,740,000 bp), and showed increased differentiation signals.

### Integration of transcriptomics and proteomics using protein-protein interaction networks

To explore the biological functions and molecular interactions in the growth plate, we added proteomics as another omics layer using liquid chromatography-mass spectrometry. This data was integrated with human orthologues of the transcriptomic data to predict protein-protein interaction (PPI) networks using network propagation analysis (see Methods). Within the resulting PPI network modules (Supplementary Data 8), we identified 122 significantly interacting proteins in AMvsAS and 129 in AMvsMA, of which 51 were common to both datasets. Similarly, in MLvsAS, we identified 123 considerably interacting proteins and 121 in MLvsMA, of which 44 interacting proteins were common between them (MLvsAS and MLvsMA). Among them, 16 proteins were found to be common in all four contrasts. Notably, GC vitamin D binding protein (*GC*) was the only one of these 16 genes, which was also identified as DEG in all four contrasts.

In addition to network propagation analysis, we investigated the post-transcriptional changes in exon usage of expressed genes by differential exon usage (DEU) analysis. In total, 6111 genes passed the threshold *p* < 0.05, and among them, 33 genes showed significant up- or downregulation ( | L2FC | > 2) in all four contrasts (Supplementary Data 9).

**Fig. 4:**
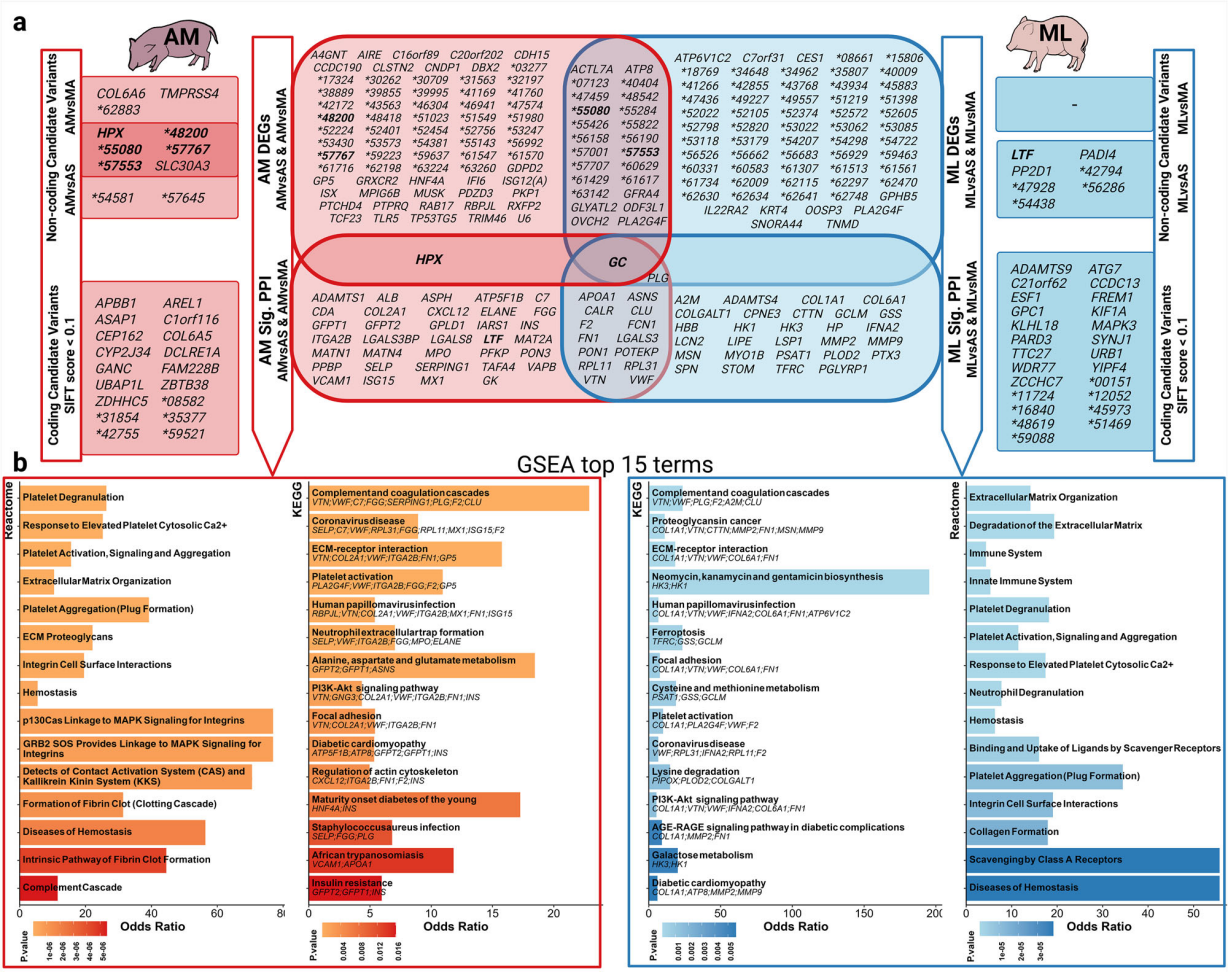
Overview of candidate gene sets. **a** Identified coding and noncoding candidate genes, DEGs, as well as PPIs, are displayed for each miniature pig breed (AM left, ML right). Common genes are shown in the middle; Genes found in multiple tests are highlighted in bold. **b** The top 15 significant terms from KEGG and Reactome pathways identified for the DEG and PPI gene-sets are shown for AM and ML. For the complete terms as well as MGI mammalian phenotype terms, please refer to the supplementary files. (*=ENSSSCG000000). n: refer to Supplementary Fig. 1. *p* < 0.05. Created using BioRender (https://BioRender.com/3qwobhv).

Finally, to prioritise biologically relevant candidates underlying miniature size, we integrated the results from the analyses described above. Candidate genes, including lncRNAs, supported by multiple lines of evidence, were considered the most likely functional candidates and are summarised in Fig. 4.

Furthermore, gene-set enrichment analysis (GSEA) provided an insight into the functional role of the identified gene sets, namely candidate variants, DEGs and PPIs. We identified 83 significant common terms between AM and ML in the KEGG pathway, Reactome, and MGI_Mammalian_Phenotype databases (Supplementary Data 10, Fig. 4b). These common terms were related to coagulation cascades and platelet functions such as “Complement and coagulation cascades”, “Platelet activation”, “Platelet Degranulation”, “Platelet Aggregation (Plug Formation)”, etc. Similarly, significant terms were found to be related to extracellular matrix, such as “ECM-receptor interaction”, “Focal adhesion”, “Regulation of actin cytoskeleton”, “Collagen Degradation”, and “Platelet Activation of Exposed Collagen”. Several immune-related terms were also detected, including “Neutrophil Extracellular Trap (NET) Formation”, which was highlighted based on candidate variants identified in ML, as well as the DEGs and PPIs dataset from AM. In addition, we found several metabolism-related terms associated with the metabolism of lipids, sugars and amino acids. Furthermore, we observed enrichment of central signalling pathways such as PI3K-Akt, MAPK, RAS and RAF2. In the MGI mammalian phenotypes database, terms related to several phenotypes, including those affecting the limb, femur, and humerus, were commonly associated with AM and ML.

## Discussion

This study presents a comprehensive multi-omics analysis of body size in pigs, with a focus on the functionally active growth plates, the central areas of longitudinal growth in long bones. The distinctiveness of this approach lies in dissecting the complex process of growth into coordinated omics layers, spanning genomic variation, chromatin architecture, gene regulation, transcriptional output, and protein abundance, in highly selected miniature pigs.

To prioritise key growth-determining factors, our strategy combined breed selection with comparable growth patterns, a focus on ROHRs specific to miniature pigs expected to harbour variants fixed by strong artificial selection, and the selection of a developmental time point reflecting growth activity while minimising major sex-related hormonal influences. We found evidence that miniature pigs exhibit steady-state growth, in contrast to the sigmoid growth curve observed in high-production pigs^26,29^. However, both AS and MA pigs were also reported to exhibit relatively steady daily weight gains over extended fattening periods, resulting in slow but uniform increases in body weight^30,31^. This fact made them suitable targets for comparison to the miniature pigs. The results of our collected measurements of the piglets were consistent with this expectation. They revealed a continuous growth pattern in both miniature and larger-sized breeds until day 80, with growth rates differing according to their final body size. Furthermore, our data suggested a significant association between metacarpal thickness and height or weight, which was in line with previous findings in Suhuai pigs, highlighting that cannon bone circumference is positively correlated with body length and body weight^32^.

These dimensional relationships were mirrored in the growth plates, where we found the length of each zone and the nuclear size of the chondrocytes corresponding to the overall size of the pig. Comparable nuclear circularity across breeds indicated that these size differences were not due to processing artefacts. This aligns with previous observations in rats, where the hypertrophic chondrocyte volume and the height of the proliferative zone correlated positively with the rate of elongation^33^. Notably, our study also revealed that the ratios of the growth-plate zones appeared to be preserved, such that the relative sizes of the zones to each other remained consistent, suggesting a strong interdependence among the zones. These observations support quantitative modulation of shared programmes rather than a change in growth-plate organisation.

Following these observations in the growth plates, we narrowed down the key factors that control local growth. Previous work has shown that growth-plate activity is influenced by both systemic signals, such as IGF-I and local regulators, including Indian hedgehog (Ihh), PTHrP, FGFs, bone morphogenetic proteins (BMPs) and vascular endothelial growth factor (VEGF) and vitamin D metabolites^34^. Given that no diminished response to growth hormone secretion was detected in miniature pigs^20,27^, we hypothesised that local control mechanisms within the growth plate are essential for the miniaturisation process. As the goal of breeding concepts in larger animal models, such as ML and AM, is to mimic the genetic structure of non-inbred populations by preserving genetic heterogeneity, while at the same time applying intense selective pressure on defining traits such as body size^35,36^, an increased likelihood of large-effect loci can be expected. Hence, our ROH analysis revealed pronounced selection footprints for ML and AM, but in distinct genomic regions, consistent with the notion that selection for small size can target breed-specific loci rather than a single shared pathway^24,25^.

In AM, a noncoding variant (rs342683522) targeting an lncRNA encoded by *ENSSSCG00000048200*, located distal to *SDR16C5*, *MOS* and *PLAG1*, emerged as one of the leading candidates. Population differentiation analyses revealed a top F_ST_ peak co-localising with this candidate variant near *MOS*/*PLAG1*, providing independent support from allele-frequency divergence. This evidence for a significant size-regulatory effect in this genomic region is consistent with various studies in human and livestock, highlighting the region of *PLAG1* to be highly associated with growth traits^37–42^. Furthermore, it was suggested that lncRNAs may contribute to distinct phenotypic and transcriptomic variation in miniature pig breeds and may drive tissue-specific regulatory programmes^43^. Thus, lncRNAs in pigs were linked to pathways related to organ size and shown to play a role in skeletal muscle development and meat production^43,44^. Accordingly, we propose that the aforementioned lncRNA, along with further differentially expressed candidates in both breeds, such as *ENSSSCG00000055080* and *ENSSSCG0000057553*, represents a major regulatory candidate for body size determination in miniature pigs.

In addition to these lncRNAs, our analysis also pointed to *HPX*, whose established role as a haem scavenger^45^ suggests a potential protective function in growth plate metabolism. This is supported by experimental evidence demonstrating that free haem directly inhibits cartilage metabolism and growth in vitro^46^. Interestingly, *HPX* has also been reported as a permeability factor in vascular tissues, where its overexpression induces angiogenesis and vascular leakage^47^. This highlights the tissue-specific role of *HPX*, suggesting that in the growth plate, it may act in a protective context by buffering haem toxicity. Additionally, it was demonstrated that *HPX* exhibits an anti-VEGF effect, a factor prominently discussed in the context of local growth plate regulation through angiogenesis, which further triggers cartilage-bone transition^47,48^. This leads to our assumption that *HPX* may be a key factor to impaired growth in the growth plates. Further evidence for the importance of haem-related processes comes from the significant enrichment of candidate variants with confirmed functional impacts on genes involved in pathways linked to platelet activation and ferroptosis, both of which are driven by haem and mediate Neutrophile Extracellular Trap formation (NETosis)^49,50^. The formation of neutrophil extracellular traps (NETs) has been shown to contribute positively to osteogenic differentiation^51^. It was interesting to note that both *LTF* and *PADI4*, two of the most prominent candidate genes in ML, are linked to NET biology. *LTF* was shown to have an inhibitory effect on the release of NETs^52^. In contrast, *PADI4* was highlighted as a driver in NET formation, promoting chromatin decondensation independent of apoptosis and reactive oxygen species (ROS)^53,54^. Furthermore, PADI4 overexpression in an osteosarcoma cell line was reported to induce NETosis, but without stimulation of cells with pro-inflammatory cytokines and bacteria^54^. Thus, this highlights the role of PADI4 as a mediator of NETosis in a non-pathological context, that can be modulated by various internal stimuli such as TNF, C5a and IL-8, homoeostatic factors like alkaline pH and hypertonicity and MAPK signalling^53,55,56^, a pathway which we also found to be enriched in our analysis. Thus, we assume that NET-related pathways, haem biology and platelet activation, which were frequently found among our common significant GSEA from DEGs and PPI networks, are important drivers of miniature size in pigs.

Moreover, ECM molecules and production were quite prominent terms in this analysis, enriched by *Fibronectin 1* (*FN1)*, *Vitronectin (VTN)* and *COL6A* family among others. ECM production plays a role in bone growth by providing a physical scaffold to control cell adhesion, differentiation, morphogenesis and homoeostasis^57^. As a major ECM protein, *VTN* was shown to have a high expression in cancellous bone and result in an increased osteoclast activity when absent in an osteoporosis mouse model^58^. Furthermore, the ubiquitous ECM glycoprotein FN1 was found to be highly expressed in resting and hypertrophic zones of growth plates^59^. It was reported that its absence leads to altered chondrogenesis as well as reduced TGFβ-mediated AKT signalling^59^. In fact, the colocalization of FN and COL6 was proposed to play a role in cell-matrix adhesion and matrix-matrix cohesion in the pericellular microenvironment of chondrocytes^60^. This pericellular matrix acts not only as a filter for the mechanical signalling in the chondrocytes but also regulates their cell size^61,62^. Subsequently, this aligns with our histological observation, as we showed that the chondrocytes of miniature pigs form smaller nuclei implying smaller cell size.

A common effect in both miniature pigs pointing to this hypothesis of reduced cell size in the growth plates contributing to miniaturisation, was highlighted by one gene, namely *GC*, which we identified in the innermost intersection of DEGs and PPI networks. Interestingly, we observed a more than 19-fold downregulation of *GC*. In line with this, a study on a patient with a homozygous deletion of *GC* and debilitating ankylosing spondylitis identified a deficiency of vitamin D-binding protein (DBP), resulting in normocalcemia but a mild disruption of bone metabolism^63^. Additionally, it was reported that the lack of vitamin D receptor in knock-out mice arrests longitudinal bone formation five weeks after birth^64^. The development and maturation of calcifying cartilage were associated with vitamin D metabolites^65^. Hence, we propose that *GC* plays an important role in local growth control as a regulatory element functioning downstream of the key variants that influence body size in miniature pigs.

## Conclusion

In summary, our data further support previous evidence pointing to the significant, though often underestimated, impact of noncoding variants in body size variation^8,11^. By integrating multiple omics layers in growth plate tissue of pigs, our study identified candidate regulatory variants and genes specifically under selection for miniature size. We propose that this dissection of the complex growth plate architecture into its functional components offers new insights into the regulatory architecture underlying skeletal growth.

## Methods

### Phenotypes and samples

Samples from 80-day-old piglets (corresponding to 5–6-year-old children^66^) from litters of two miniature breeds, namely Aachen Minipig (AM) and Mini-LEWE (ML), as well as two larger pig breeds, Angeln Saddleback (AS) and Mangalitza (MA), were obtained (Sampling strategy: see Supplementary Fig. 1). These piglets and their littermates were kept in the same stables, received the same food type and amount (relative to their body weight), and were measured every seven days starting shortly after birth or after weaning for three parameters, namely body height (floor to shoulder level - cm), thickness of their metacarpal bone on the right forelimb (cm) and weight (kg). The number of measured piglets varied with the size of each litter: AS = 9, MA = 4, AM = 4 and ML= variable (9 for weight and 4 for other parameters due to access restrictions). On day 80 after birth, four piglets of each litter, two male and two female, underwent general anaesthesia by intramuscular injection of Azaperone (Stresnil, 2 mg per kg; Elanco GmbH, Germany) and Ketamine (Ketamidor, 20 mg per kg; Richter Pharma AG, Austria) and were euthanised by intracardial application of T61 (tetracaine hydrochloride, mebezonium iodine & embutramide cocktail, 6 ml per 50 Kg, MSD Tiergesundheit - Intervet Deutschland GmbH, Germany). After confirmation of successful euthanasia, a piece of ear cartilage or skin was sampled for WGS sequencing. From the right limbs, distal growth plates of the femur and humerus of each pig were fixed in Bouin’s solution to be embedded in paraffin for histological studies.

The distal growth plates of the left femur and humerus were sampled for omics analyses using 1 mm disposable biopsy punches (Kai Medical, Germany). Each biopsy was punched according to a predefined order along the growth plate line, from left to right, and labelled by position, region, and planned sequencing method. The reason was to ensure that each biopsy corresponded to the analogous position and anatomical region in all sampled piglets. The designated biopsies for each omics method were flash frozen or immediately processed according to the subsequent protocols. This procedure was approved by the animal welfare officer of the University of Veterinary Medicine Hannover (“Tötungsanzeige”, ID TIHO-T-2019-12 and TIHO-T-2020-9), in accordance with national and international guidelines. We have complied with all relevant ethical regulations for animal use.

### Univariate and multivariate linear mixed models for growth traits

We applied the Multivariate Covariance Generalised Linear Model (McGLM) framework^67^ to analyse the development of piglet growth traits. McGLM enables the joint modelling of correlated responses, which in our case arise from the repeated measures structure of the data. This multivariate approach allows estimation of growth trait correlations while simultaneously accounting for covariates and within-individual dependencies.

A challenge in the analysis was the uneven data coverage. On some days, the number of available measurements was reduced due to limited access to the animals caused by maternal behaviour. Additionally, the miniature pig breeds contributed fewer observations overall. To address these limitations, we first fitted univariate linear mixed models for each trait, including day, sex, breed, and the breed-by-day interaction as fixed effects. Female sex and the AM breed were set as reference categories. The dependency structure was modelled by specifying both an independent and a compound symmetry residual component.

For the multivariate analysis, we grouped breeds by size, defining larger-sized breeds (AS, MA) and miniature breeds (AM, ML). This grouping improved coverage across days and provided a higher number of observations within each size category. To account for the increased heterogeneity introduced by this grouping, we additionally specified a random slope and a random intercept–slope interaction component. For the AS breed, which had sufficient observations, we applied the multivariate model to estimate within-breed correlations between growth traits. These estimates were then compared to the breed-adjusted correlations from the size-grouped analysis. The dependency structure was specified as described above, taking into account repeated measures and individual variation. Model performance was assessed using residual diagnostics as well as coefficients of determination (R² and adjusted R²).

All analyses were conducted in Python 3.9.6. Data wrangling and visualisation were performed with pandas v.1.5.3^68^, NumPy v.1.23.4^69^, matplotlib v.3.7.0^70^, and seaborn v.0.13.2^71^. Model fitting was carried out using the mcglm v.0.2.4 package^72^, a Python implementation of the multivariate covariance generalised linear model framework. ChatGPT was used to assist with code refinement for multivariate analysis and plots.

### Histological analysis

Sampled growth plates from the right humerus and femur of 16 piglets (four piglets per breed) were decalcified in EDTA solution (pH 7.2, Carl Roth, Karlsruhe, Germany) at 37 °C for four weeks with daily change of the solution. Next, the samples were embedded in paraffin, sectioned to a thickness of 2-3 μm, and stained using the standard Haematoxylin and Eosin (H&E) staining protocol. The samples were scanned using the fully automated scanning system Zeiss Axio Scan Z1 (Zeiss, Oberkochen, Germany). We chose a magnification of 10 on a Plan-Apochromat numerical aperture 0.45 M27 objective, a Hitachi HV-F203SCL colour camera in 1 × 1 binning mode, and a 1x camera adaptor. This optical setup resulted in a lateral (xy axes) pixel resolution of 0.441 µm per pixel. Each image comprised about 400–4000 million single pixels per tissue. In total, we acquired 82 tissue sections from all four pigs (8–15 sections per location per breed), of which six images were blurry and removed from further analysis.

Before further analysis, we defined the epiphyseal region of the growth plate in the long bones and cropped it accordingly. To measure and compare the thickness of the growth plates in the miniature and larger-sized pig breeds, the inherently characteristic accordion-like structure of the growth plate needed to be virtually reshaped (or reconstructed) into a linear structure. Thus, the cropped images were straightened using a macro (custom script) written for FIJI’s open-source image processing software^73^. Within the macro, a user interface guided the experimenter in a double-masked manner through the data folders. It allowed them to draw a line, straightening the image to a line thickness of 500 pixels. Therefore, any technical (sample stretching, squeezing due to processing or sectioning) or biological (tiny curvatures) characteristics of tissues were compensated for, allowing for an objective quantification. Next, the thickness of the four central regions of the growth plates—resting, proliferative, hypertrophic, and mixed zones—was measured based on their pixel distance using five iterations of random lines in all tissues. The whole process was performed three times to ensure the randomness of the drawn lines.

### Tissue classification

The following described processing and analysis routines were all carried out in the ZEN 3.5 environment, utilising three modules: Intellesis, Image Analysis, and Open Application Development. The acquired tissue images were binned to 1% of their original size by custom macros. Subsequently, within the downsampled images, we classified all pixels by an a priori trained pixel classifier to automatically identify tissue compartments and to isolate the cartilage zone of the growth plate for single-cell analysis. After the classification, we upsampled the images and fused them with their originals. Thereby, we generated a resource for the following image analysis. Briefly, a hierarchical image analysis identified the cartilage zone using a binary classification map. Within this first class, the HE-stained growth columns were identified, and herein, single nuclei were observed. We extracted colour proportions, morphometrics, localisations, and quantities from all identified objects.

In the next step, we used a linear mixed-effects model (lmer, lmerTest package v.3.1-3 and lme4 package v. 1.1-33^74^ in R environment) to test the differences in extracted nuclei parameters among all four breeds. Next, we performed a pairwise comparison among all breeds. The obtained *p-values* were adjusted with the Bonferroni-Holm correction method.

### Whole genome sequencing data processing

Whole genome sequencing (WGS) data from a total of 109 pigs were obtained, including eight miniature pig breeds/populations and 18 larger-sized breeds. Sequencing data were retrieved from previous studies^13,75^ or provided at the National Centre for Biotechnology Information (NCBI) Sequence Read Archive (SRA), as detailed in Supplementary Data 1. In addition, for WGS, one individual was selected from each breed that was sampled in this study (sequencing data for ML and AM were already used for a previous study^75^ and published at NCBI SRA). For these samples, libraries were prepared using the KAPA HyperPrep Kit (Hoffmann-La Roche, Basel, Switzerland) according to the manufacturer’s instructions. They were sequenced on an Illumina NovaSeq 6000 (Illumina, San Diego, CA, USA) in paired-end mode with a read length of 2 × 150 bp. Datasets were submitted to the NCBI SRA (PRJNA635602).

All sequencing data were quality-controlled, further processed, and mapped using our WGS pipeline with parameters described in detail in our previous publication^13^. Processed reads were aligned to the autosomal *Sus scrofa 11.1* reference genome (Ensembl) using the Burrows-Wheeler Aligner (BWA) v. 0.7.17-r1188^76^. Duplicate reads were flagged using Picard tools v. 3.1.0^77^ and the final dataset was indexed.

### Runs of homozygosity analysis

#### Generation of a genomic variant set for ROH detection

To detect runs of homozygosity (ROHs) for the miniature pigs of interest (AM and ML), we selected a subset of 55 pig samples from 20 populations (see Supplementary Data 1) meeting our criteria for a reliable SNP-set with sufficient variation for ROH calling: Those samples were selected, which had a sequencing coverage of 12x to 49x, for a minimum of three individuals per breed to provide statistical robustness and allow breed-specific comparisons. This selection process prioritised samples with consistent and sufficient coverage to minimise incomplete genotyping due to low sequencing depth and subsequent higher false positive rates for ROH calling. Variant calling and filtering of these data were performed according to our established ROH-detection strategy^13^ using the Genome Analysis Toolkit (GATK) v. 4.1.9.0^77^ tools, including BaseRecalibrator, HaplotypeCaller, BaseQualityScoreRecalibrator, and CalculateGenotypePosteriors. Functional annotation of variants was performed using SNPEff v4.3 t, build 2017-11-24^78^. After variant calling, all variants located on mitochondrial DNA (MT), sex chromosomes (X and Y) and unplaced scaffolds were removed using vcftools v. 0.1.15^79^. Indels, multiallelic sites, and variants with a minor allele count of less than one or a base quality score of minQ 30, variants with read depths outside the range 6–100 or sites with more than 41 missing genotypes were removed from the analysis (-minDP 6 -maxDP 100 -max-missing-count 41).

#### Calling runs of homozygosity

Based on the obtained SNP set, we called ROHs for AM and ML using the workflow established in our previous study^13^. This workflow integrates the rule-based method implemented in PLINK v. 1.90b6.21^80^ with the Hidden Markov Model (HMM)-based algorithm in RZooRoH v. 0.3.1^81^.

To ensure accurate ROH detection, we optimised the ROH calling parameters based on the guidelines from our previous study. We applied the suggested simultaneous ROH detection using a rule- and model-based caller, followed by a merging step for ROHR^13^. We employed a sliding window approach with dataset-specific adjustments based on SNP density, heterozygosity, and sequencing depth, for the rule-based method in PLINK. Sliding window size (homozygous-window-snp) and scanning window threshold (homozygous-window-threshold) were calculated using previously validated formulae to ensure sensitivity and specificity, allowing detection of even small ROHs arising from historical recombination events dating back to the domestication of pigs approximately 10,000 years ago^82^.

We applied two custom parameter sets for ROH calling to maximise detection accuracy. The first parameter set (named PLINK_A, analogous to what was used in the published workflow) was designed to detect shorter ROHs with a minimum length of 20 SNPs. In this configuration, we set the minimum ROH length (homozyg-kb) to 1.2 kb, the minimum number of SNPs per ROH (homozyg-snp) to 20, the minimum SNP density (homozyg-density) to 0.06 SNPs per kb, the sliding window size (homozyg-window-snp) to 20 SNPs, and the scanning window threshold (homozyg-window-threshold) to 0.25. The second parameter set (named PLINK_B) was optimised to detect longer ROHs with a minimum length of 130 SNPs. Here, the minimum ROH length (homozyg-kb) was set to 7.8 kb, the minimum number of SNPs per ROH (homozyg-snp) to 130, the minimum SNP density (homozyg-density) to 0.06 SNPs per kb, the sliding window size (homozyg-window-snp) to 130 SNPs, and the scanning window threshold (homozyg-window-threshold) to 0.03.

Furthermore, we applied a model-based approach using the hidden Markov model (HMM) implemented in RZooRoH. Given that the domestication of pigs started about 10,000 years ago, corresponding to 5000 pig generations with a generation interval of 2 years, we set the upper limit of the recombination rate (RK) to 10,000, reflecting the oldest detectable homozygous by descent (HBD) segments. We defined a mixKR model with eight predefined HMM classes, using the median RK values estimated from the KR model: 18, 28, 74, 169, 468, 1002, 3062 and 10,000. A genotyping error rate of 0.25% was also applied. ROHs were assigned to HBD or non-HBD regions using the Viterbi algorithm, ensuring robust classification based on recombination history.

#### Runs of homozygosity region detection

Finally, overlapping ROHs for all individuals within one miniature pig breed of interest (AM or ML), as well as ROHs overlapping across both miniature pig breeds, AM and ML, were merged using a custom R script to identify ROH regions (ROHRs) for each dataset and tool as described before^13^. Finally, the resulting ROHRs were collected into a single file using bedtools v. 2.30.0^83^, thereby generating a comprehensive ROHR dataset for use in downstream analyses.

### Chromatin conformation and accessibility

#### Hi-C sequencing and data analysis

We performed high-throughput chromatin conformation capture using the Hi-C technique to study genome-wide chromatin interactions within the nucleus of a bone-marrow-derived mesenchymal stem cell (MSC)-sample isolated from one of the studied ML piglets from the hip area^84^. The library preparation method was adapted from a Hi-C protocol for eukaryotic genomes^85^: MSCs were detached from their culture vessel using TrypLE Express Enzyme (Gibco, Massachusetts, USA), immediately fixed in 2% formaldehyde for 10 minutes at room temperature and lysed for another 10 min on ice. The chromatin was cleaved using DpnII exonuclease (NEB, Ipswich, MA, USA) and sheared in a Covaris M220 (Covaris Company, Massachusetts, USA) with the following settings: peak power 30.0, duty factor 10.0, cycles/burst 200, and duration 60 s. Next, the library was prepared using the NEBNext Ultra II DNA Library Prep Kit for Illumina and NEBNext Multiplex Oligos for Illumina (NEB). The libraries were sequenced at 2 × 150 bp on an Illumina NextSeq 2000. After sequencing, the fastq files were processed, and reads were mapped to the pig reference genome Sus scrofa 11.1^86^ using the FAN-C pipeline v.0.9.26^87^ with BWA v.0.7.17^76^. After mapping, filtering and deduplication, replicates were combined. Read pairs were then used to generate binned and KR-normalised Hi-C maps. Using a window size of 10, topologically associating domains (TADs) were detected using TopDom v.0.0.228 on 50-kb binned and KR normalised maps^88^.

### ATAC sequencing and peak calling

Open chromatin regions (OCRs) were identified using the Assay of Transposase Accessible Chromatin sequencing (ATAC-seq; *n* = 24: 12 piglets sampled from humerus and femur each) according to a previously published protocol^89^: Briefly, nuclei were extracted from snap-frozen growth plate tissues in ice-cold lysis buffer containing Nonidet P40 substitute (ThermoFisher, Massachusetts, USA) as its primary lysis reagent. Genomic DNA was cleaved with Tn5 transposase (Illumina) as described in the Omni-ATAC protocol^89^. Cleaved DNA fragments were purified using the MiniElute PCR Purification kit (Qiagen) and indexed using Unique Dual Indexes (IDT, Iowa, USA). Libraries were amplified using NEBNext High-Fidelity 2x PCR Master Mix (NEB) and sequenced with 2 × 100 bp on an Illumina NovaSeq 6000 (Supplementary Data 1).

Sequencing reads were trimmed to remove the adaptor sequences using Cutadapt v.4.6^90^ and mapped to the *Sus scrofa* reference genome using Bowtie v.2.5.0^91^. SAMtools v.1.19.2^92^ was used for filtering, sorting, and duplicate removal. Biological replicates from forelimb and hindlimb tissues of the same breed were pooled using SAMtools v.1.19.2^92^. Coverage tracks for sequencing reads were generated with deepTools v.3.5.1^93^. ATAC-seq peaks were then identified from the merged alignments using Genrich v.0.6.1^94^.

### RNA sequencing and transcriptome profiling

The freshly sampled biopsy punches from distal growth plates from the left humerus and femur of 3 piglets per miniature pig breed AM and ML, as well as per large breed AS and MA (*n* = 24), were incubated for 90 min in DMEM solution with 0.1% collagenase type A (Sigma-Aldrich, St. Louis, USA). Subsequently, RNA isolation, library preparation and sequencing were performed as previously described^95^. In total, 23 of 24 sampled biopsy punches had RNA integrity numbers greater than 7 and were used for library preparation. One sample from MA did not meet the quality criteria and was thereby not further processed. The prepared libraries were sequenced on an Illumina NovaSeq 6000 for 2 × 100 bp (Supplementary Data 1).

Quality control, data processing and the detection of counts were performed according to our previously published approach^95^. All 23 samples were fitted into a negative binomial generalised linear model matrix based on breed, sampling location (humerus or femur) and sex, using DESeq2 v.1.32.0^96^. The variables sex and sampling location were included for batch correction. Fitted data were used to call the differentially expressed genes (DEGs) based on variable breed, with one miniature breed (AM or ML) contrasted against one large breed (MA or AS) as a control for each test. The exploratory thresholds for selecting DEGs were set to an absolute Log2FoldChange (|L2FC | ) > 2 and *p* < 0.05. This threshold was used to define the ‘candidate gene set’ for downstream analyses. Among these, genes with padj < 0.05 were additionally highlighted as FDR-controlled findings. Subsequently, candidate genes were not interpreted directly based on differential expression alone but were carried forward into subsequent analyses and further prioritised by incorporating independent genomic evidence.

Furthermore, to detect exon-level regulatory changes not captured by gene-level expression analyses, we performed DEU analysis using mapped RNA-seq reads with DEXSeq v.4.50.0^97^, as described previously^84^. DEXSeq fit the exon counts into a negative binomial generalised linear model^84^. This matrix model was specified in R-syntax by R-syntax “~ sample + exon + breed:exon”, thereby accounting for sample-specific effects and testing differential exon usage throughout the interaction between all breeds and exon. Hence, the model accounted for repeated measures in each sample and a (breed x exon) interaction. Additional covariates such as sex or sampling location were not included in the model, as the analysis focused on breed-associated exon usage patterns and inclusion of further variables would have substantially increased model complexity relative to the available sample structure. The threshold for selecting differentially used exons was set to *p* < 0.05 and |L2FC | > 2. Exons with padj < 0.05 were additionally highlighted as FDR-controlled findings. The resulting set of candidate exons was subsequently overlapped with candidate variants to assess the potential effects of coding or noncoding variants on post-transcriptional regulation.

Codes for plotting the statistical output were refined partially with the assistance from AI-tool Copilot.

### Detection and prioritisation of candidate variants

#### Variant calling

The complete dataset of 109 porcine whole genome sequencing data (Supplementary Data 1) was used to identify candidate variants within ROHRs. SNPs and small INDELs were detected using GATK tools, v.4.1.9.0^77,98^. Structural variants were detected using two complementary callers, namely Manta v.1.6.0^99^ and Lumpy^100^ implemented through the Smoove v.0.2.8 pipeline^101^. The resulting structural variant calls were integrated into a unified dataset using SURVIVOR v.1.0.7^102^, which merged overlapping variants and accounted for small breakpoint differences between callers. The merged dataset was subsequently re-genotyped using SVTyper v.0.7.1^103^. To avoid sex bias, all tools were applied to autosomal chromosomes only. Structural variants larger than 1 Mb were excluded to minimise the number of false positives associated with repetitive genomic regions or assembly uncertainties following the initial detection process. In addition, variants with low genotype quality or insufficient read support were filtered out following the initial detection process. The resulting high-confidence variant dataset was subsequently used for downstream analyses.

#### Genotype filtering and filtering for a variant subset

First, a subset of variants, including SNPs, INDELs, and structural variants, that overlapped with ROHRs identified in the miniature pigs (AM, ML, or both, AM + ML) was extracted. Then, this subset of variants was further filtered to retain only variants with homozygous alternative genotypes (‘1/1’) in the miniature pig breeds AM, ML, or both. In contrast, a wild-type genotype (‘0/0’) was required in all larger pig breeds, using bcftools v.1.19^104^. Genotypes from miniature pig breeds other than AM and ML were not restricted by genotype filtering in this analysis.

In addition, all coding homozygous variants within ROHR were annotated using ENSEMBL VEP release 110^105^. Variants with a SIFT score < 0.1 were further considered as potential candidate variants.

#### Data integration and functional annotation of noncoding variants

We overlapped all filtered homozygous variants with the open chromatin regions (OCR) identified by ATAC-Seq peaks for functional annotation. Target genes were mapped to regulatory elements indicated by ATAC-seq peaks using the generalised Activity-By-Contact (gABC) model implemented in STARE v.1.0.4^106^. Contact frequencies derived from Hi-C data together with chromatin accessibility signals (read counts) from ATAC-seq peaks were used to predict regulatory element-target gene interactions. Interactions with a gABC score > 0.02 were retained.

In the next step, the subset of variants meeting the criteria mentioned above in the filtering procedure was tested for overlap with predicted regulatory elements (ATAC peaks) and their target genes using the gABC model. Only those variants were considered as potential noncoding candidate variants, which could be allocated by this method to a DEG in one of the miniature pig breeds (or both). In addition to that, we tested the structural variants obtained from the genotype and ROHR-filtering step for a direct overlap with DEGs of at least one bp using bcftools v.1.19^104^.

### Transcription factor footprinting

To further prioritise regulatory variants, we used TF-footprinting information. For this approach, peak calling for ATAC-seq data was performed on each merged alignment of the analysed pig breeds using MACS2 v.2.2.7.1 with the parameters “--nomodel --shift -100 --extsize 200 --broad”^[ 107^ and Genrich v.0.6.1^94^. The resulting peaks were merged using bedtools v.2.30.0^83^. Transcription factor footprints were predicted using the TOBIAS v.0.13.3 pipeline^108^. Only motifs corresponding to TFs with a mean normalised count (ratio of medians) gene expression value greater than ten in any of the replicates (see the transcriptome analysis) were retained for further study. Finally, the subset of variants of interest was investigated for TF binding at predicted footprint loci using SNPScan, implemented in the Java package ape v.3.0.6^109^. SNPs with a fold-change in binding affinity greater than five or less than 0.2 between the reference and alternative alleles were considered to have a significant effect on TF binding.

### Assigning F_ST_-regions and noncoding variants to GWAS-genes

Independent of ROH analysis, we estimated fixation indices (F_ST_) using vcftools (v. 0.1.16) based on our SNP-dataset, called from 109 pig samples^104^ to investigate the genetic differentiation between miniature and larger-sized pig breeds using only biallelic sites. Furthermore, in order to evaluate the robustness of the observed differentiation signals in a broader population context, F_ST_ analysis was extended to a panel of 201 pigs using additional data from Sequence Read Archive (SRA) including 23 larger-sized pigs and 70 miniature pigs mainly designated as Chinese local breeds. Filters were applied to exclude indels, enforce a minor allele frequency (MAF) threshold of 0.05, and remove sites with more than 20% missing genotypes (Max Missing = 0.8). Mitochondrial, sex and unplaced chromosomes were also excluded from the analysis.

F_ST_ values were calculated per site and using sliding windows with a window size of 50 kb and a step size of 10 kb. To determine the significance of genetic differentiation, we calculated F_ST_ thresholds based on the top 0.2% of the distribution, corresponding to weighted F_ST_ values of ≥ 0.57054 for windowed F_ST_s for the discovery panel of 108 pigs and ≥ 0.57127 for the expanded panel of 201 pigs. Manhattan plots were generated using Haploview v.4.2 to visualise these results^110^. Genes and variants with high F_ST_ values above the threshold were identified and extracted for further analysis. The number of samples did not allow for an exclusive F_ST_ analysis for both miniature pigs.

To explore the potential overlap between genes associated with ‘height’ identified in human GWAS studies and expressed genes from our porcine transcriptomic dataset, we retrieved all the genes related to the search term ‘height’ from the NHGRI-EBI GWAS Catalogue^111^. We relied on the GWAS Catalogue’s predefined genome-wide significance threshold, which was applied during data curation. Pig orthologous genes were identified using the Ensembl BioMart tool v.2.60.1^112,113^, which maps human genes to their pig counterparts based on conserved gene annotations. Gene symbols, Ensembl gene IDs and cross-references to the NCBI gene database were used to ensure accurate ortholog mapping. We then screened all genes expressed in our transcriptomic dataset for overlap with porcine orthologs identified in the GWAS catalogue. Genes were considered to be expressed if the sum of their normalised raw counts was greater than one across all biological replicates.

### Mass Spectrometry-based proteomics

Flash-frozen samples of growth plates from the left humerus, in biological triplicates, were immersed in cold lysis buffer (Tris-HCl 50 mM, NaCl 150 mM, 1% NP-40, 0.5% Sodium deoxycholate, 1% SDS, and Protease Inhibitor Mix 1%), homogenised in a pre-cooled SpeedMill, and immediately centrifuged at 4 °C. The samples were left on ice for 30 min before they were centrifuged at 4 °C. An aliquot from the supernatant (lysate) was taken for measuring the protein concentration using a BCA assay. 20 μg of protein were taken from the lysate for precipitation in ice-cold acetone (final concentration of 80%). Proteins were spun down at 11,000 × *g* and 4 °C for 15 min, reduced, alkylated and digested with Trypsin Gold (Promega) following the manufacturer’s instructions. Next, samples were purified using StageTips following the protocol of Rappsilber et al.^114^. Purified peptides were dried in a SpeedVac Vacuum Concentrator and resuspended in MS buffer (0.1% formic acid, 5% acetonitrile in water (MS grade).

Next, samples were analysed via liquid chromatography–mass spectrometry using a Vanquish Neo nanoflow UHPLC System coupled to an Orbitrap Eclipse mass spectrometer (Thermo Scientific) as previously described^115^. In brief, 500 ng peptides were separated on a reversed phase nanoViper™ PepMap™ column with a gradient of buffer A (0% ACN, 0.1% FA) and B (80% ACN, 0.1% FA). Peptides were ionised with a Nano Spray Flex Ion Source and stainless-steel emitters. Precursor scans were performed in the Orbitrap mass analyser. The 20 most intensive precursors were submitted to HCD fragmentation and fragments were scanned in the linear ion trap.

MS raw data were processed using MaxQuant software (version 2.4.14.0) for the identification and quantification of proteins, employing preconfigured settings. Additionally, label-free quantification via LFQ and IBAQ was selected, and mzTab tables were written^115^. MS spectra were searched against the UniProt database of porcine proteins (ID: UP000008227, all entries, downloaded on 21 June 2024).

### Network propagation with protein-protein interactions

To integrate the transcriptomics and proteomics data for each pairwise comparison of miniature and larger pigs and to map the data onto networks, we applied a workflow based on three steps: (i) network generation, (ii) node initialisation, and (iii) network propagation.

For *network generation*, we utilised an integrated, high-quality human protein-protein interaction (PPI) network provided by the ConsensusPathDB resource^116^, which was compiled from 18 different public repositories. While the ConsensusPathDB contains different heterogeneous interactions, we focused on PPIs that were experimentally measured and evaluated with a quality score that is composed of annotation-based and topology-based measures to quantify the confidence associated with each given interaction (range [0,1]). For our study, we retained only interactions with a confidence score greater than 0.9, resulting in a PPI network comprising 10,534 proteins and 137,509 interactions.

For *node initialisation*, we computed for each pig gene, *g*_*i*_, a combined transcriptomics and proteomics relevance score by

$${S}_{i}={S}_{i,t}+{S}_{i,p}$$

where ${S}_{i,t}$ and ${S}_{i,p}$ are the scores from the RNA-seq and proteomics data analysis for each pairwise comparison:

$${S}_{i,t}=\left|{\log }_{10}{p}_{i,t}\right|\left|{\log }_{2}{r}_{i,t}\right|$$

$${S}_{i,p}=\left|{\log }_{10}{p}_{i,p}\right|\left|{\log }_{2}{r}_{i,p}\right|$$

Here, ${p}_{i,t}$ and ${p}_{i,p}$ are the P-values computed for gene/protein *g*_*i*_ in the comparison of miniature and larger pigs with transcriptomics and proteomics data, respectively, and ${r}_{i,t}$ and ${r}_{i,p}$ are the corresponding fold-changes. ${S}_{i,t}$ P-values and fold-changes were computed with DESeq2 and ${S}_{i,p}$ with an unpaired Welch t-test. Network nodes were initialised with the relevance scores of their homologous pig genes using the UniProt annotation UP000008227, if both measurements for transcriptomics and proteomics were available (ca. 2400 genes/proteins in the four different comparisons). All other network nodes were set to zero.

For *network propagation*, to derive network modules that aggregate most of the relevance associated with the observed differences between miniature and large pigs, the weights of the initialised nodes were distributed across the entire network using the NetCore programme^117^, which employs the novel concept of node coreness. After the re-ranking process, nodes were compared against random graph permutations to identify nodes that were significantly re-ranked. Then, significantly re-ranked nodes were connected with the top-scored nodes, and the derived connected components built the final network modules. The entire workflow was applied to four comparisons involving miniature and larger pigs (AMvsAS, AMvsMA, MLvsAS, and MLvsMA), resulting in four distinct sets of network modules.

### Gene-set enrichment and functional analysis

Functional annotation and gene-set enrichment analysis were performed separately on different data sets using the enrichR tool (R package, version 3.4^118,119^) for the “KEGG_Human_2021”, “Reactome_Pathway 2024”, and “MGI_Mammalian_Phenotype_Level_4_2024” databases. The first gene set consisted of a list of genes assigned to both coding and noncoding variants in each of the miniature pig breeds. The second set analysis was performed on the gene list obtained from DEGs and protein-protein interaction networks. As these gene lists derived from our data integration approach were relatively small, enrichment results were initially filtered using a nominal *p*-value threshold (*p* < 0.05). Adjusted *p*-values reported by Enrichr are provided additionally. Significant terms among all the different datasets were checked for overlapping events.

### Statistics and reproducibility

Next generation sequencing data were analysed using established and widely used bioinformatics workflows. Statistical analyses, including differential expression analysis, enrichment analysis, and network propagation, were performed using the methods and thresholds described in the corresponding sections of the Methods. Reproducibility was supported by the use of biological replicates across multiple individuals per litter and by performing analyses across different pig breeds.

### Ethics approval and consent to participate

The euthanasia of the pigs was approved by the animal welfare officer of the University of Veterinary Medicine Hannover (“Tötungsanzeige”, IDs TIHO-T-2019-12 and TIHO-T-2020-9), in accordance with national and international guidelines. The pigs’ owner, the Institute of Animal Genomics, agreed on the use of the samples for this study.

## Supplementary information

Transparent Peer Review file

Supplementary Tables 1-12 X Figure 1

Description of Additional Supplementary Materials

Supplementary Data 1

Supplementary Data 2

Supplementary Data 3

Supplementary Data 4

Supplementary Data 5

Supplementary Data 6

Supplementary Data 7

Supplementary Data 8

Supplementary Data 9

Supplementary Data 10

Supplementary Data 11

nr-reporting-summary-updated

## Data Availability

All nucleic acid and RNA sequencing data were submitted to NCBI SRA (PRJNA635602, PRJNA1274638, PRJNA1200293, see also detailed information in Supplementary Data 1). Proteomics data have been deposited to the ProteomeXchange Consortium via the PRIDE^120^ partner repository with the dataset identifier PXD069390. All numerical data underlying the plots in Figs. 1b–d and 2c, d are provided in Supplementary Data 11.
