# Supplementary material for: Integrative multi-omics analysis of growth plate regulation underlying body size in miniature pigs
Source: Commun Biol. 2026 Jun 30;9:875. doi: 10.1038/s42003-026-10538-9 (PMC13315324; doi:10.1038/s42003-026-10538-9)
Supplement: Supplementary file 2 — Supplementary Tables 1-12 X Figure 1 [file 42003_2026_10538_MOESM2_ESM.pdf]

Supplementary Tables containing details of multivariate analysis.

Supplementary Table 1. Sample description by breed

Breed	n	Observations	Male	Female	Day range
AM	4	20	2 (50%)	2 (50%)	47-80
AS	9	108	2 (22.2%)	7 (77.8%)	2-78
MA	4	25	3 (75%)	1 (25%)	37-80
ML	5	27	2 (40%)	3 (60%)	5-61

Supplementary Table 2. Sample description by breed size

Breed	n	Observations	Male	Female	Day range
Large	13	133	5 (38.5%)	8 (61.5%)	2-80
Mini	9	47	4 (44.4%)	5 (55.6%)	5-80

Supplementary Table 3. Descriptive statistics by breed

Breed	Trait	Mean ± SD	Minimum	Median	Maximum
AM	Height	25.85 (3.10)	20.00	26.50	31.00
AM	Weight	6.40 (2.10)	3.51	6.33	10.19
AM	Metacarpal thickness	7.25 (0.55)	6.00	7.00	8.00
AS	Height	29.46 (8.11)	12.00	30.00	43.00
AS	Weight	8.29 (4.70)	0.71	10.00	19.00
AS	Metacarpal thickness	9.33 (1.88)	5.00	10.00	12.50
MA	Height	33.46 (2.43)	29.00	33.50	39.00
MA	Weight	12.58 (3.20)	8.20	12.00	19.50
MA	Metacarpal thickness	10.24 (0.77)	8.50	10.00	11.50
ML	Height	23.20 (3.81)	13.00	24.00	27.50
ML	Weight	4.10 (1.55)	0.79	04.02	6.88
ML	Metacarpal thickness	6.70 (0.99)	5.00	7.00	8.00

Note: SD = standard deviation

Supplementary Table 4. Descriptive statistics by breed size

Breed	Trait	Mean ± SD	Minimum	Median	Maximum
Large	Height	30.21 (7.54)	12.00	32.00	43.00
Large	Weight	9.61 (4.67)	0.71	10.80	19.50
Large	Metacarpal thickness	9.50 (1.76)	5.00	10.00	12.50
Mini	Height	24.33 (3.73)	13.00	25.00	31.00
Mini	Weight	5.08 (2.12)	0.79	4.75	10.19
Mini	Metacarpal thickness	6.94 (0.87)	5.00	7.00	8.00

Note: SD = standard deviation

Supplementary Table 5. Regression parameter estimates from univariate mixed models.

Term	Height [95% CI]	Weight [95% CI]	Metacarpal thickness [95% CI]
Intercept	11.91*** [7.19, 16.62]	−4.81** [−8.24, −1.37]	4.67*** [3.07, 6.27]
Sex [male]	0.11 [−1.14, 1.36]	0.73 [−0.39, 1.86]	0.27 [−0.13, 0.68]
Breed [AS]	4.88* [0.09, 9.67]	6.48*** [2.97, 9.99]	1.86* [0.24, 3.48]
Breed [MA]	13.90*** [7.96, 19.83]	8.97*** [4.64, 13.31]	3.22** [1.21, 5.23]
Breed [ML]	3.05 [−2.16, 8.26]	4.90* [1.07, 8.72]	−0.36 [−2.13, 1.40]
Days	0.22*** [0.15, 0.30]	0.17*** [0.12, 0.23]	0.04** [0.02, 0.06]
Days × Breed [AS]	0.10** [0.02, 0.17]	0.01 [−0.05, 0.06]	0.03* [0.01, 0.06]
Days × Breed [MA]	−0.10* [−0.19, −0.01]	−0.04 [−0.11, 0.02]	−0.00 [−0.03, 0.03]
Days × Breed [ML]	−0.02 [−0.10, 0.07]	−0.08** [−0.14, −0.02]	0.02 [−0.01, 0.05]
R^2	0.90	0.86	0.85
Adjusted R^2	0.90	0.85	0.84

Note: Female sex and the AM breed are set as reference categories. Estimates are shown with 95% confidence intervals. Coefficient of determination (R^2) and adjusted R^2. Significance: *** $p < 0.001$, ** $p < 0.01$, * $p < 0.05$.

Supplementary Table 6. Estimated dispersion parameters from univariate mixed models.

Component	Height	Weight	Metacarpal Thickness
τ_0 (Independent)	3.51 [2.67, 4.34] ***	1.76 [1.22, 2.29] ***	0.41 [0.30, 0.51] ***
τ_1 (Compound Symmetry)	1.30 [0.25, 2.36] *	1.20 [0.35, 2.06] **	0.13 [0.02, 0.24] *

Note: τ_0 corresponds to the independent residual variance (matrix Z_0), τ_1 corresponds to the compound symmetry covariance (matrix Z_1). Significance levels: *** $p < 0.001$, ** $p < 0.01$, * $p < 0.05$.

Supplementary Table 7. Regression parameter estimates from multivariate mixed model.

Term	Height (95% CI)	Weight (95% CI)	Metacarpal Thickness (95% CI)
Intercept	16.83 [16.07, 17.59]***	1.67 [1.33, 2.01]***	6.60 [6.30, 6.91]***
Sex [male]	-0.30 [-1.39, 0.80]	0.25 [-0.28, 0.78]	0.02 [-0.30, 0.34]
Breed Size [Small]	-1.69 [-3.80, 0.42]	-1.88 [-3.02, -0.73]***	-1.82 [-2.60, -1.05]***
Days	0.30 [0.29, 0.32]***	0.17 [0.16, 0.19]***	0.068 [0.063, 0.073]***
Days × Breed Size [Small]	-0.12 [-0.16, -0.07]***	-0.07 [-0.10, -0.04]***	-0.026 [-0.039, -0.012]***
R^2	0.87	0.85	0.83
Adjusted R^2	0.86	0.84	0.83

Note: Coefficients reported with 95% confidence intervals. Significance: *** $p < 0.001$

Supplementary Table 8. Estimated dispersion parameters from multivariate mixed model.

Component	Height	Weight	Metacarpal Thickness
τ_0 (Independent)	5.04 [3.94, 6.14] ***	2.00 [1.26, 2.75] ***	0.57 [0.46, 0.67] ***
τ_1 (Compound Symmetry)	-0.30 [-1.37, 0.76]	-0.38 [-0.60, -0.16] **	0.05 [-0.18, 0.28]
τ_2 (Slope variance)	0.00 [0.00, 0.00]	0.00 [0.00, 0.00]	0.00 [0.00, 0.00]
τ_3 (Intercept–Slope)	0.02 [0.003, 0.04] *	0.008 [0.002, 0.01] *	0.001 [-0.003, 0.005]

Note: τ_0 = independent residual variation (Z_0), τ_1 = compound symmetry (Z_1), τ_2 = slope variance (Z_2), τ_3 = intercept - slope covariance (Z_3). Significance: *** $p < 0.001$

Supplementary Table 9. Estimated trait correlations from multivariate mixed model.

Trait Pair	ρ	95% CI	Significance
Height – Weight	0.38	[0.24, 0.51]	***
Height – Metacarpal Thickness	0.62	[0.54, 0.71]	***
Weight – Metacarpal Thickness	0.57	[0.45, 0.69]	***

Note: ρ represents the estimated correlation between trait pairs after accounting for covariates and random effects. 95% confidence intervals are shown in brackets. Significance: *** $p < 0.001$

Positive correlations indicate that individuals with higher values in one trait tend to have higher values in the other trait, conditional on the modelled effects.

Supplementary Table 10. Regression parameter estimates for AS breed from multivariate mixed model.

Term	Height (95% CI)	Weight (95% CI)	Metacarpal Thickness (95% CI)
Intercept	16.73 [15.90, 17.56]***	1.74 [1.44, 2.05]***	6.68 [6.30, 7.05]***
Sex [male]	0.56 [-0.96, 2.08]	0.09 [-0.40, 0.58]	0.17 [-0.35, 0.70]
Days	0.32 [0.31, 0.34]***	0.16 [0.14, 0.18]***	0.07 [0.07, 0.08]***
R^2	0.90	0.79	0.77
Adjusted R^2	0.90	0.79	0.77

Note: Coefficients reported with 95% confidence intervals.

Significance: *** $p < 0.001$

Supplementary Table 11. Estimated dispersion parameters for AS breed from multivariate mixed model.

Component	Height	Weight	Metacarpal Thickness
τ_0 (Independent)	5.22 [4.15, 6.30] ***	3.13 [2.08, 4.18] ***	0.93 [0.75, 1.11] ***
τ_1 (Compound Symmetry)	-0.12 [-1.23, 0.99]	-0.77 [-0.88, -0.66] ***	0.09 [-0.13, 0.31]
τ_2 (Slope variance)	0.00 [-0.00, 0.00]	0.00 [0.00, 0.00]	-0.00 [-0.00, 0.00]
τ_3 (Intercept–Slope)	0.03 [0.02, 0.05] ***	0.01 [0.01, 0.02] ***	0.003 [-0.001, 0.007]

Note: τ_0 = independent residual variation (Z_0), τ_1 = compound symmetry (Z_1), τ_2 = slope variance (Z_2), τ_3 = intercept - slope covariance (Z_3). Significance: *** $p < 0.001$

Supplementary Table 12. Estimated trait correlations for AS breed from multivariate mixed model.

Trait Pair	ρ	95% CI	Significance
Height – Weight	0.49	[0.39, 0.59]	***
Height – Metacarpal Thickness	0.72	[0.63, 0.80]	***
Weight – Metacarpal Thickness	0.72	[0.59, 0.86]	***

Note: ρ represents the estimated correlation between trait pairs after accounting for covariates and random effects. 95% confidence intervals are shown in brackets. Significance: *** $p < 0.001$

Positive correlations indicate that individuals with higher values in one trait tend to have higher values in the other trait, conditional on the modelled effects.

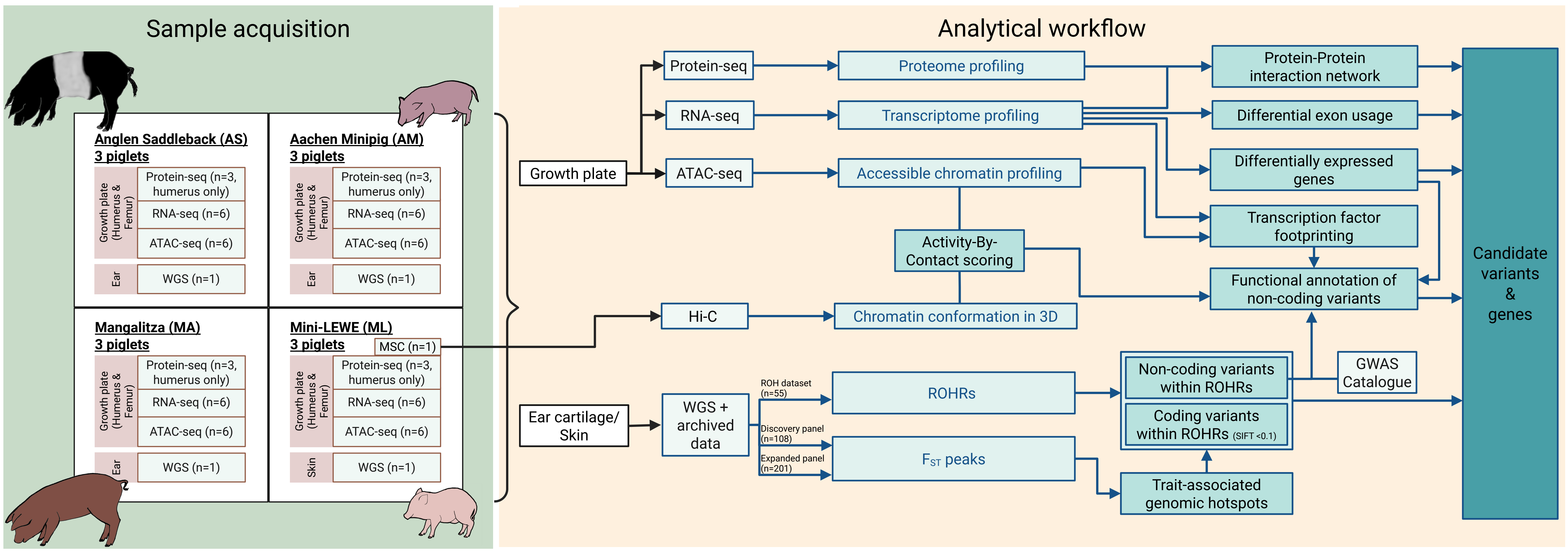

Supplementary Figure 1. Overview of collected samples, sequencing methods and analytical workflow for the identification of candidate genes and variants. Created in BioRender (https://BioRender.com/doplhdf).
