# Supplementary material for: Integrative multi-omics analysis of growth plate regulation underlying body size in miniature pigs
Source: Commun Biol. 2026 Jun 30;9:875. doi: 10.1038/s42003-026-10538-9 (PMC13315324; doi:10.1038/s42003-026-10538-9)
Supplement: Supplementary file 3 — Description of Additional Supplementary Materials [file 42003_2026_10538_MOESM3_ESM.pdf]

Description of Additional Supplementary Files

File name: Supplementary Data 1

Description: Summary of sequencing data used for this study.

File name: Supplementary Data 2

Description: Runs of homozygosity regions (ROHRs) and variants called for miniature pigs of interest. Chromosome, start-end position and length of the ROHRs are provided for Aachen minipig (AM), Mini-LEWE (ML) and the combined dataset of both of them (AM+ML) as well as the number of homozygous alternative SNPs in each breed within ROHRs.

File name: Supplementary Data 3

Description: Overview of candidate variants. All variants within ROHRs meeting the genotype filtering criteria for the minipigs are given. Coding variants were assigned as "candidate variants" when having a SIFT score of <0.1. As filtering criteria for non-coding variants, they were assigned as "candidate non-coding variants" if they were located in a chromatin-contact region assigned to a DEG by gABC model. No structural variants were found meeting these criteria. In addition, transcription factor footprinting results for the non-coding candidate variants and predicted altered transcription factor binding affinities are displayed.

File name: Supplementary Data 4

Description: Differentially expressed genes (DEGs) in the minipigs of interest. Common DEGs above the significance threshold of absolute Log2FoldChange (|l2fc|) of 2 and a p-value < 0.05 as well as human ortholog names are given. In addition, DEGs resulting from contrasts of one of the miniature breeds, Aachen minipig (AM) or Mini-LEWE (ML), against one large breed Mangalitza (MA) or Angeln Saddleback (AS) are displayed. Genes with significant FDR correction (padj < 0.05) are highlighted in bold, and additionally underlined when common across different tests.

File name: Supplementary Data 5

Description: Hi-C sequencing statistics and gABC-predicted regulatory element-gene interactions. Hi-C sequencing and mapping statistics are reported from the mesenchymal stem cell sample of a Mini-LEWE pig, including sequencing output, library characteristics, and mapping metrics (valid read pairs, PCR duplicates, and cis/trans contact proportions). This file also provides the predicted regulatory element–target gene interactions for the four analysed pig breeds obtained using the generalised Activity-By-Contact (gABC) model implemented in STARE, which integrates ATAC-seq activity with Hi-C chromatin contact frequencies.

File name: Supplementary Data 6

Description: Manhattan plot and output details of FST. Overview of the identified FST windows above the 0.2% distribution threshold corresponding to weighted FST ≥ 0.57054 in

108 pig WGS data (first table) and weighted FST ≥ 0.57127 in 201 pig WGS data (second table). Chromosomes, start and end of the binned windows as well as genes in designated windows are shown.

File name: Supplementary Data 7

Description: GWAS-genes linked to non-coding variants associated with height. Summary of variants from the filtered-subset within ROHRs, meeting the following criteria: Located within a predicted functional element (ATAC-peak), assigned to a gene by gABC model, which is expressed in the growth plate, and found in the GWAS catalogue. Transcription factor (TF) binding activities were tested for this list of variants. In addition, structural variants from the filtered subset overlapping with GWAS-expressed genes are displayed.

File name: Supplementary Data 8

Description: List of proteins and protein-interactions with a combined confidence score >0.9. Source databases, interaction partners (genes) and interaction confidence scores are displayed.

File name: Supplementary Data 9

Description: Differentially Exon Usage (DEU) across all four pig breeds. The table displays significant exon usage (p-value < 0.05) in annotated genes (Ensembl ID) and exons (exon ID), followed by base mean and dispersion for each sample, normalized counts in each breed and the log2FC. Additionally, genomic data, gene names and type as well as the raw counts are shown. Exons and genes with significant FDR correction (padj < 0.05) are highlighted in bold.

File name: Supplementary Data 10

Description: Complete list of significant terms (p-value < 0.05) enriched from KEGG, Reactome as well as MGI_Mammalian phenotype. Four tables are provided: (1) enrichment analysis of genes derived from differentially expressed genes (DEGs) and protein–protein interaction (PPI) network analyses for the AM breed; (2) the same analysis for the ML breed; (3) enrichment analysis of genes associated with coding and non-coding candidate variants identified in AM; and (4) the corresponding analysis for ML. For each term, the number of overlapping genes, p-value, adjusted p-value, combined score, and associated gene list are reported. Terms passing false discovery rate correction (FDR-adjusted p-value < 0.05) are highlighted in bold.

File name: Supplementary Data 11

Description: Numerical data source for plots in figures 1b-d and 2c-d
