# Supplementary material for: Integrative multi-omics analysis of growth plate regulation underlying body size in miniature pigs
Source: Commun Biol. 2026 Jun 30;9:875. doi: 10.1038/s42003-026-10538-9 (PMC13315324; doi:10.1038/s42003-026-10538-9)
Supplement: Supplementary file 1 — Transparent Peer Review file [file 42003_2026_10538_MOESM1_ESM.pdf]

nature portfolio

Peer Review File

# Integrative multi-omics analysis of growth plate regulation underlying body size in miniature pigs

Corresponding Author: Professor Julia Metzger

This manuscript has been previously submitted at another journal. This document only contains information relating to versions considered at Communications Biology.

**This file contains all reviewer reports in order by version, followed by all author rebuttals in order by version.**

Version 0:

Reviewer comments:

Reviewer #1

(Remarks to the Author)

464/5000
Body size is definitely worth the attention of scientific researchers, especially when it comes to humans. Among domestic animals, the size of the body directly affects the income of the breeder. For instance, pigs may also be donors of xenogeneic organs, which is of even greater significance. However, the sample size of the experimental animals, pigs, in this study makes it difficult to gain sufficient confidence in the conclusion of this research. The multi-omics data integration analysis also appears overly ordinary, and the excessive grouping leads to particularly redundant content. A comprehensive reanalysis may be very necessary.

Main:

Structural variation detection utilized the results of manta and smoove software, but the results of structural variation were separate and needed to be integrated into a single dataset. In addition, the accuracy of variant detection using second-generation resequencing data may be less than 70%. Only the insertion and deletion phenotypes are good, and necessary quality control is required.

2. On line 727, change "ML" to "MA".

In the study, pigs were divided into four groups, resulting in a very small sample size for each group. Perhaps it would be more appropriate to divide it into two groups: small pigs and large pigs. The structure of research papers will be clearer, more credible and easier to understand.

4. Do RNA-seq and ATAC-seq come from the same individual? This is very important.

5. Were these 24 individuals used for resequencing?

6. More miniature pigs, such as those from local Chinese breeds, can be included, which will make the Fst analysis more robust.

7. The scale of resequencing is only about 100 heads. Is it representative enough to conduct ROH-related analysis?

Reviewer #2

(Remarks to the Author)
COMMSBIO-25-11001-T review: “Integrative multi-omics analysis of growth plate regulation underlying body size in miniature pigs”
This study aimed to characterize bone growth regulation through an integrated multi-omics approach, specifically targeting

the growth plate. It combines histological analysis with genetic and genomic techniques to identify genomic regions that regulate growth, potentially explaining variations in pig height.
The question of genetic regulation of growth has been the subject of numerous studies, the main result of which has been the identification of a large number of genes, each with a limited phenotypic effect. In this study, the authors used a multi-omics approach combined with sharp tissue targeting to collect the portion of bone involved in growth. Combined with the choice of pig lines of different sizes, notably minipigs, this ensured the reliability of the experiment.
The basis of the study was to identify ROH regions common to the two minipig breeds as candidate regions to find genes and genetic variants that may explain the difference in size between the minipig breeds and larger pigs. Additional omics, such as RNA-seq and ATAC-seq, as well as variants identified across breeds, help focus on the genomic regions of interest. The combination of these methods allows candidate genes for growth to be identified. In this context, the authors identified two lncRNA and three genes that may play a central role in the growth plate.
The methods sound correctly detailed. However, some specific points regarding the experimental design need to be clarified.
The most critical finding is that it is difficult to follow the experimental design and find information in the supplementary files. I suggest adding a diagram to explain the process and correctly identify the nine supplementary files. Currently, it is necessary to open all of them to find the right one. Moreover, another difficult issue to follow is the exact number of animals/samples used, while sometimes another number of samples is declared in the supplementary files. Another difficult aspect was to distinguish between their own data and public data, and to determine what had been obtained from the same samples.
Line 546: The exact number of samples at each stage and for each measure should be given in the experimental design.
Line 741: Identifying DEGs without corrected p-values is not acceptable. This was done for exon usage (line 748). In the second model (exon usage), the tissue (humerus vs femur) and sex were not included. Probably, there are limitations to statistics due to the design (2 or 3 samples per breed/tissue). Please clarify. The same for enrichment analysis (line 899). Then, in Results, line 261, it is not an adjusted p-value.
Line 864: Where can we find the list of genes and proteins used to construct the network? I think I understood later on, but could you please clarify what you did at the beginning? If I understand correctly, you retrieved a complete network from the database before selecting the nodes in subsequent steps.
Where in the text and how were these results (network) used? Maybe I missed the information.
What was the added value of performing DEU compared to DEG in your analysis?
Where are the results of the HiC analysis? How were these data used? It is not clear.
The relevant results are probably buried in the list of results.
It would be useful to add a paragraph explaining why the authors decided to focus on these results (genes and lncRNA), as the information comes as somewhat of a surprise in the discussion. Maybe at the end of the Results section with Figure 3. It might be useful to have a schema showing the relevant functional results.
We would also be grateful if you could comment on the appropriateness and validity of any statistical analysis, as well the ability of a researcher to reproduce the work, given the level of detail provided.
The conclusion proposed by the authors is perhaps overly general.
Minor comments
Line 56: Which lncRNA?
Please avoid the abbreviation 'L2FC' in the legend of Figure 3b.
According to supplementary data 2, there are 76 large and 32 small animals (1 NA), whereas lines 209–211 state that there are 65 large, 32 small, and 12 crossbred animals.
Line 106: How many litters?
Line 305: Supplementary data 6, there is only 135 and not 136 lines. The results for SSC18 is missing.
Line 568: Please explain these numbers.
Line 588: Where does the classifier come from?
Line 598: I assume this was done with R. Please specify.
Lines 687-705: Please give the number of samples obtained from how many animals for HiC. Same question for ATACseq (Lines 707-716).
Line 780: How many samples per analysis?
Line 830: Please clarify, three independent samples or three samples from the same humerus?
Lines 860-861: ConsensusPathDB-human is not only a PPI network but also “genetic, metabolic, signaling, gene regulatory and drug-target interactions, as well as biochemical pathways”. This is important unless the authors have chosen a subset of information to construct the networks. Please clarify. The same for line 333.
Line 880: How many nodes were kept at this stage? Is it the 2400?
Line 901-905: Please check what is declared. This is the method section.
Line 920: I wasn't able to access the PXD069390 dataset. It might be useful to provide a direct link to it.
Supplementary data 7: What do the genotypes in columns AG to EK correspond to?

Reviewer #3

(Remarks to the Author)
The authors have used an integrative approach, combining fine phenotyping, anatomical/histological studies and multi-omics analyses, to identify genes/variants regulating body size in miniature pigs. The topic is of interest to a wide scientific community.
The authors have performed an impressive piece of work, generating loads of different data. Each analysis is well done and described.
However the current version of the manuscript is more like a catalogue of candidate genes and the authors don't discuss the

potential molecular mechanisms or how those identified genetic variants might impact body size. In addition, no molecular functional validation is done. I thus found some of the conclusions quite speculative. For example, how the noncoding variant targeting the lncRNA ENSSSCG00000048200 impact body size?
I therefore recommend a major revision of the manuscript.

I've few questions:
1-I wonder why the ROH analyses were only done for the 2 miniature pig breeds and not also for the 2 large sized pig breeds. Human selection might have induced ROHR associated with body size in large sized pigs, as well.
2- I worry that by focussing on variants/genes only found in ROHR identified in miniature pigs, the authors might be missing other variants associated with body size.
3- The authours didn't use the eQTL data from the FarmGTEx consortium. It'll be interesting to see if any of the identifed variants are also cis or trans eQTL SNPs.
4- I don't see what brings the exon usage analysis. How does it help in identifying the variants/genes associated with body size in miniature pigs? Could you explain please?

I've few comments:
1- Please had the rsID for the variants when possible.
2- Please rephrase L315: "homozygous mutant variant (g.75646178-75646178:G>A)" as "homozygous alternative SNP (rsID xx, g.75646178:G>A)"
3- Be careful in the use of mutant instead of alternative allele.
4- Please mentioned the variants (rsID and/or chromosome/position/alleles) when discussed (i.e. L418).

Version 1:

Reviewer comments:

Reviewer #1

(Remarks to the Author)
Overall, after a round of meticulous revisions, I have no further doubts about this research and recommend its acceptance and publication.

Reviewer #2

(Remarks to the Author)

I congratulate the authors on their careful revision of the manuscript and on their responses to my comments. These extra details might help readers to understand the experiment, the results and why they are interesting.
The additional figure (supplemental data 12), which summarises the data usage in the study, is greatly appreciated.
To provide greater transparency and rigour, the number of samples and animals utilised across the various sections of the manuscripts is now documented.
It was also observed that supplementary information regarding adjusted p-values across various statistics utilised in multiomics studies had been incorporated, despite the challenges encountered in their acquisition (small sample size).
Thank you for explaining in the manuscript the interest in finding DEGs versus DEUs.

Reviewer #3

(Remarks to the Author)
I'm OK with the final version of the manuscript. The authors have answered all my points. However I still believe that looking at the results of the pigGTEx consortium might have give extra insights on the results, despite not including eQTL data for growth plate tissue.

**Open Access** This Peer Review File is licensed under a Creative Commons Attribution 4.0 International License, which permits use, sharing, adaptation, distribution and reproduction in any medium or format, as long as you give appropriate credit to the original author(s) and the source, provide a link to the Creative Commons license, and indicate if changes were made.
In cases where reviewers are anonymous, credit should be given to 'Anonymous Referee' and the source.
The images or other third party material in this Peer Review File are included in the article's Creative Commons license, unless indicated otherwise in a credit line to the material. If material is not included in the article's Creative Commons license and your intended use is not permitted by statutory regulation or exceeds the permitted use, you will need to obtain permission directly from the copyright holder.
To view a copy of this license, visit https://creativecommons.org/licenses/by/4.0/

**Reviewers Response Letter**

We thank the reviewers for their careful evaluation of our manuscript and for their constructive comments, corrections, and justified concerns. In the following, we address each comment and revision point in detail.

Reviewer #1 (Remarks to the Author):

--------------------------------------

Body size is definitely worth the attention of scientific researchers, especially when it comes to humans. Among domestic animals, the size of the body directly affects the income of the breeder. For instance, pigs may also be donors of xenogeneic organs, which is of even greater significance. However, the sample size of the experimental animals, pigs, in this study makes it difficult to gain sufficient confidence in the conclusion of this research. The multi-omics data integration analysis also appears overly ordinary, and the excessive grouping leads to particularly redundant content. A comprehensive reanalysis may be very necessary. Answer: We thank the reviewer for highlighting the importance of body size research and for the constructive comments regarding sample size, the multi-omics integration, and the structure of the analyses. We carefully reconsidered these aspects and performed additional analyses to strengthen the robustness of our conclusions. All changes and additional analyses are described in detail in the responses below and have been incorporated into the revised manuscript.

Main:

1. Structural variation detection utilized the results of manta and smoove software, but the results of structural variation were separate and needed to be integrated into a single dataset. In addition, the accuracy of variant detection using second-generation resequencing data may be less than 70%. Only the insertion and deletion phenotypes are good, and necessary quality control is required.

Answer: We thank the reviewer for raising these important points regarding the detection and filtering of structural variants. Structural variants were identified using two complementary callers, Manta and Smoove, which rely on different signals from short-read sequencing data and therefore increase sensitivity for different classes of structural variants. As described in the Methods, the resulting calls were integrated using SURVIVOR, which merges overlapping variants while accounting for small breakpoint differences between callers. The merged structural variant set was subsequently re-genotyped using SVTyper to obtain consistent genotype information across all samples. This multi-tool strategy is further supported by recent benchmarking in pigs showing that SV caller choice substantially influences the resulting callsets; that WGS-based callsets, particularly Manta, retain moderate similarity to long-read-based callsets, and that short-read WGS performs more effectively for deletions than for insertions (He et al. iScience. 2025;28(3):111983).

We clarified this in the methods section- Line 806-811: "Structural variants were detected using two complementary callers, namely Manta v.1.6.0 and Lumpy implemented through the Smoove v.0.2.8 pipeline. The resulting structural variant calls were integrated into a unified

dataset using SURVIVOR v.1.0.7, which merged overlapping variants and accounted for small breakpoint differences between callers. The merged dataset was subsequently re-genotyped using SVTyper v.0.7.1."

Furthermore, to ensure a high-confidence dataset, several quality control steps were applied. Analyses were restricted to autosomal chromosomes to avoid sex-related biases, and variants larger than 1 Mb were excluded to minimize potential false positives arising from repetitive genomic regions or assembly uncertainties. Following variant detection and genotyping, low-confidence variants were removed, resulting in a filtered structural variant dataset used for downstream analyses. To clarify these procedures, we have expanded the description of the structural variant detection and filtering steps in the revised Methods section in lines 8114-817: "To avoid sex bias, all tools were applied to autosomal chromosomes only. Structural variants larger than 1 Mb were excluded to minimise the number of false positives associated with repetitive genomic regions or assembly uncertainties following the initial detection process. In addition, variants with low genotype quality or insufficient read support were filtered out following the initial detection process. The resulting high-confidence variant dataset was subsequently used for downstream analyses..."

2. On line 727, change "ML" to "MA".

Answer: Amended.

3. In the study, pigs were divided into four groups, resulting in a very small sample size for each group. Perhaps it would be more appropriate to divide it into two groups: small pigs and large pigs. The structure of research papers will be clearer, more credible and easier to understand.

Answer: We thank the reviewer for this thoughtful suggestion. We agree that grouping pigs into "small" and "large" categories may appear conceptually simpler. However, miniature pig breeds have been developed independently through selective breeding, and different genetic variants and gene pathways may underlie their reduced body size. Analyzing the miniature breeds separately therefore allows the identification of breed-specific selection signals that might otherwise be masked if all small breeds were pooled into a single group.

To account for both possibilities, we designed our analysis in two steps. First, breed-specific comparisons were performed to identify genomic regions associated with body size within each miniature pig breed. Second, we conducted analyses combining the miniature pig breeds to identify shared genetic signals associated with reduced body size across breeds. This approach allows us to capture both breed-specific and common genetic mechanisms underlying miniature size. We have clarified this in the revised manuscript to improve readability and better explain the study design, see lines 112-116: "As the two miniature pig breeds were developed in different breeding programmes and may therefore harbour distinct genetic variants underlying reduced body size, analyses were first conducted separately to identify breed-specific genomic signals before evaluating shared signatures across miniature pigs."

4. Do RNA-seq and ATAC-seq come from the same individual? This is very important.

Answer: Yes, this is correct; RNA-seq and ATAC-seq data were obtained from the same individuals and the same sampling locations. To facilitate readability, we added **Supplementary Data 12**, which provides an overview of all collected samples and sequencing approaches, including the number of animals and samples.

5. Were these 24 individuals used for resequencing?
Answer: In our study, we investigated 12 individuals (forelimb and hindlimb from each individual = 24). From these individuals, we selected from each breed of sampled piglets one individual for whole genome sequencing. To improve clarity regarding the sample number, we added **Supplementary Data 12**, which provides an overview of the collected samples and sequencing approaches, and revised the corresponding sentence in the Methods section.
Line 645-647: "In addition, for WGS, one individual was selected from each breed that was sampled in this study (sequencing data for ML and AM were already used for a previous study and published at NCBI SRA)."

6. More miniature pigs, such as those from local Chinese breeds, can be included, which will make the Fst analysis more robust.
Answer: We appreciate the reviewer's suggestion. In addition to our original FST study, we did a follow-up analysis extended to an expanded dataset including in a total of 201 pigs with 23 additional larger-sized pigs and 70 additional miniature pigs mainly defined as Chinese local breeds. The following findings were made:
Line 345-353: "In a second step, the FST analysis was extended to an expanded dataset comprising 201 pigs, including additional miniature pig populations and further larger-sized breeds for comparison, to evaluate the robustness of the observed differentiation signals in a broader population context. In this extended analysis, most of the differentiation peaks identified in the initial analysis were no longer detected above the significance threshold. However, the two peak regions with 349 FST window hits on chromosomes three and four remained significant, with slightly shifted genomic positions (chromosome 3: 38,710,001-43,790,000 bp; chromosome 4: 75,630,001- 82,740,000 bp), and showed increased differentiation signals."
Line 861-864: "Furthermore, in order to evaluate the robustness of the observed differentiation signals in a broader population context, FST analysis was extended to a panel of 201 pigs using additional data from Sequence Read Archive (SRA) including 23 larger-sized pigs and 70 miniature pigs mainly designated as Chinese local breeds."
Supplementary Data 7 has been updated accordingly.

7. The scale of resequencing is only about 100 heads. Is it representative enough to conduct ROH-related analysis?
Answer: We thank the reviewer for raising this important point. Runs of homozygosity (ROH) analyses have been widely applied in livestock genomics. They are per definition contiguous homozygous genomic segments within an individual genome and can therefore be detected at the level of single individuals. Population sample size primarily affects the ability to compare

ROH patterns across individuals or populations rather than the detection of ROHs themselves. That is why ROH analyses for breed-specific traits can be reliably performed with moderate sample sizes, particularly in domesticated populations where strong artificial selection and relatively recent breed formation often result in long and detectable ROH segments. Previous studies have successfully identified ROH patterns and selection signatures in livestock populations using comparable or even smaller sample sizes (e.g., Gertrud Grilz-Seger et al, 2018, https://doi.org/10.1016/j.jevs.2018.09.004; G. Schiavo et al, 2021, https://doi.org/10.1111/age.13045).

In the present study, 109 individuals were analyzed across different breeds, which is within the range commonly used for ROH-based analyses in livestock populations. Moreover, the focus of our study was the identification of genomic regions specifically enriched with ROH in miniature pig breeds compared with larger pigs, rather than the estimation of population-level inbreeding coefficients. The clear ROH signals observed in our dataset are consistent with strong artificial selection for body size in miniature pig breeds. We clarified this point in the revised manuscript.

Line 73-75: These contiguous homozygous segments arise from recent selection or shared ancestry and are therefore powerful indicators of genomic regions affected by breeding[14,15].

Reviewer #2 (Remarks to the Author):

--------------------------------------

COMMSBIO-25-11001-T review: "Integrative multi-omics analysis of growth plate regulation underlying body size in miniature pigs"

This study aimed to characterize bone growth regulation through an integrated multi-omics approach, specifically targeting the growth plate. It combines histological analysis with genetic and genomic techniques to identify genomic regions that regulate growth, potentially explaining variations in pig height.

The question of genetic regulation of growth has been the subject of numerous studies, the main result of which has been the identification of a large number of genes, each with a limited phenotypic effect. In this study, the authors used a multi-omics approach combined with sharp tissue targeting to collect the portion of bone involved in growth. Combined with the choice of pig lines of different sizes, notably minipigs, this ensured the reliability of the experiment.

The basis of the study was to identify ROH regions common to the two minipig breeds as candidate regions to find genes and genetic variants that may explain the difference in size between the minipig breeds and larger pigs. Additional omics, such as RNA-seq and ATAC-seq, as well as variants identified across breeds, help focus on the genomic regions of interest. The combination of these methods allows candidate genes for growth to be identified. In this context, the authors identified two lncRNA and three genes that may play a central role in the growth plate.

The methods sound correctly detailed. However, some specific points regarding the experimental design need to be clarified.

1. The most critical finding is that it is difficult to follow the experimental design and find information in the supplementary files. I suggest adding a diagram to explain the process and correctly identify the nine supplementary files. Currently, it is necessary to open all of them to find the right one. Moreover, another difficult issue to follow is the exact number of animals/samples used, while sometimes another number of samples is declared in the supplementary files. Another difficult aspect was to distinguish between their own data and public data, and to determine what had been obtained from the same samples.

Answer: Thank you for this helpful suggestion. We agree that improving the clarity of the study design and the organisation of the supplementary material is important. To facilitate navigation and improve transparency, we added **Supplementary Data 12**, which provides a comprehensive overview of all analysed samples, including the number of animals, the biological material collected, and the sequencing approaches applied to each sample. In addition, we revised the corresponding descriptions in the Methods and Results sections to ensure that the reported sample numbers are consistent throughout the manuscript.

See for example lines 744-746: "Open chromatin regions (OCRs) were identified using the Assay of Transposase Accessible Chromatin sequencing (ATAC-seq; n=24: 12 piglets sampled from humerus and femur each) according to a previously published protocol."

2. Line 546: The exact number of samples at each stage and for each measure should be given in the experimental design.

Answer: We have included the number of the measured piglets for the multivariate analysis in our method section.

Line 541-543: "The number of measured piglets varied with the size of each litter: AS=9, MA=4, AM=4 and ML= variable (9 for weight and 4 for other parameters due to access restrictions). "

Additionally, we summarized the number of obtained samples for each step of data analysis in a figure provided as Supplementary Data 12.

3. Line 741: Identifying DEGs without corrected p-values is not acceptable. This was done for exon usage (line 748).

Answer: We thank the reviewer for this important comment and agree that controlling for multiple testing is the standard practice in differential expression analyses. In this study, our intention was to perform an exploratory analysis aimed at identifying candidate genes that may contribute to body size differences when integrated with additional genomic information, including enhancer activity, chromatin accessibility, and variants located within regulatory elements. Importantly, candidate genes were **not interpreted solely based on differential expressions** but were further prioritised by integrating independent genomic evidence across these datasets. Because these downstream analyses rely on combining multiple layers of evidence, we initially reported genes passing a nominal p-value threshold together with an absolute $\log_2$ fold-change filter to avoid prematurely excluding candidates with potentially subtle but biologically relevant expression changes.

Due to the limited sample size and the resulting reduced statistical power, applying strict FDR thresholds alone resulted in very few detectable DEGs. However, we agree that reporting adjusted p-values is important for transparency and for allowing readers to evaluate the robustness of the results. In response to the reviewer's suggestion, we now provide FDR-adjusted p-values for all genes in **Supplementary Data 5**, and we explicitly indicate which genes meet the conventional significance threshold (padj < 0.05). This allows readers to clearly distinguish between FDR-supported findings and exploratory candidates used for the integrative analyses. We have also clarified this point in the Methods section:

Lines 777-784: "The exploratory thresholds for selecting DEGs were set to an absolute Log2FoldChange (|L2FC|) > 2 and p-value < 0.05. This threshold was used to define the 'candidate gene set' for downstream analyses. Among these, genes with padj < 0.05 were additionally highlighted as FDR-controlled findings. Subsequently, candidate genes were not interpreted directly based on differential expression alone but were carried forward into subsequent analyses and further prioritised by incorporating independent genomic evidence."

Furthermore, we now report the number of genes with padj < 0.05 in the Results section to ensure transparency:

Lines 273-278: "In total, 96 differentially expressed genes (DEGs) were called, including 57 lncRNAs, pseudo or novel genes passing the significance threshold in both contrasts AMvsAS and AMvsMA, of which eight DEGs passed the corrected FDR threshold (padj < 0.05). Additionally, 86 DEGs (68 were either lncRNA, pseudo- or novel genes) were identified in the

contrasts MLvsAS and MLvsMA, of which 47 DEGs passed the corrected FDR threshold (Fig. 3b-c, Supplementary Data 5)."

4. In the second model (exon usage), the tissue (humerus vs femur) and sex were not included. Probably, there are limitations to statistics due to the design (2 or 3 samples per breed/tissue). Please clarify.

Answer: We thank the reviewer for raising this important point. Differential exon usage was analysed using the generalized linear model (GLM) framework implemented in DEXSeq. The model was specified to test the primary biological contrast of interest. Additional variables such as sex or tissue of origin were not included, as they were not part of the tested contrast and including them would substantially increase model complexity relative to the available sample structure. In small datasets, adding multiple covariates can lead to unstable parameter estimates and reduced statistical power. We have clarified the model specification in the Methods section.

Lines 785-795: "Furthermore, to detect exon-level regulatory changes not captured by gene-level expression analyses, we performed DEU analysis using mapped RNA-seq reads with DEXSeq v.4.50.097, as described previously84. DEXSeq fit the exon counts into a negative binomial generalized linear model84. This matrix model was specified in R-syntax by R-syntax "~ sample + exon + breed:exon", thereby accounting for sample-specific effects and testing differential exon usage throughout the interaction between all breeds and exon. Hence, the model accounted for repeated measures in each sample and a (breed x exon) interaction. Additional covariates such as sex or sampling location were not included in the model, as the analysis focused on breed-associated exon usage patterns and inclusion of further variables would have substantially increased model complexity relative to the available sample structure."

Furthermore, in **Supplementary Data 10**, we highlighted exons and genes with significant FDR correction (padj < 0.05) in bold.

5. The same for enrichment analysis (line 899). Then, in Results, line 261, it is not an adjusted p-value.

Answer: Enrichment analysis was performed using the Enrichr platform. Because the gene sets analysed for enrichment were derived from variant-based candidate filtering, the resulting lists contained only a small number of genes. For such small gene sets, enrichment analyses often have limited statistical power and strict filtering based solely on multiple-testing–adjusted p-values may lead to very few or no enriched terms being detected. Therefore, enrichment results were initially filtered using a nominal significance threshold ($p < 0.05$) to identify potentially relevant functional categories. However, in response to the reviewer's suggestion and to ensure transparency, the adjusted p-values reported by Enrichr are now highlighted in the revised supplementary table (**Supplementary Data 11**) and referenced in the Results section, allowing readers to distinguish between exploratory findings and results that remain significant after multiple-testing correction.

See lines 963-967: “As these gene lists derived from our data integration approach were relatively small, enrichment results were initially filtered using a nominal p-value threshold ($p < 0.05$). Adjusted p-values reported by Enrichr are provided additionally. Significant terms among all the different datasets were checked for overlapping events.”

6. Line 864: Where can we find the list of genes and proteins used to construct the network? I think I understood later on, but could you please clarify what you did at the beginning? If I understand correctly, you retrieved a complete network from the database before selecting the nodes in subsequent steps.

Answer: Thank you for this comment; We totally agree. First, we constructed a complete human PPI network from experimentally derived PPI public resources and in the next step we used orthologs information to initialize nodes in the network with proteomic and transcriptomic data of homologous pig genes (UniProt annotation UP000008227). These node weights were then distributed across the network with network propagation in order to identify subnetworks (pathways, functions) that are mainly affected by the data.

The PPI network was integrated from different human resources, each PPI was experimentally measured and evaluated with topological and annotation-based confidence scores and the final PPI network was constructed from PPIs with a combined confidence score >0.9 (Kamburov & Herwig, 2022; reference 115). This led to a human PPI comprising 10,534 proteins and 137,509 interactions. The list of proteins and PPIs can be found in **Supplementary Data 9** and is also retrievable from the ConsensusPathDB website.

Lines 922-930: “For network generation, we utilised an integrated, high-quality human protein-protein interaction (PPI) network provided by the ConsensusPathDB resource[116], which was compiled from 18 different public repositories. While the ConsensusPathDB contains different heterogeneous interactions, we focused on PPIs that were experimentally measured and evaluated with a quality score that is composed of annotation-based and topology-based measures to quantify the confidence associated with each given interaction (range [0,1]). For our study, we retained only interactions with a confidence score greater than 0.9, resulting in a PPI network comprising 10,534 proteins and 137,509 interactions.”

7. Where in the text and how were these results (network) used? Maybe I missed the information.

Answer: The network was used to integrate proteomics with transcriptomics data at the network level, and results were presented in subsection “Integration of transcriptomics and proteomics using protein-protein interaction networks”. We added a reference to the Methods section at the respective position in the text to make the link clearer.

See lines 356-362: “To explore the biological functions and molecular interactions in the growth plate, we added proteomics as another omics layer using liquid chromatography-mass spectrometry. This data was integrated with human orthologues of the transcriptomic data to predict protein-protein interaction (PPI) networks using network propagation analysis (see Methods). Within the resulting PPI network modules (Supplementary Data 9), we identified

122 significantly interacting proteins in AMvsAS and 129 in AMvsMA, of which 51 were common to both datasets."

8. What was the added value of performing DEU compared to DEG in your analysis?
Answer: We thank the reviewer for raising this important point. We have clarified in the revised manuscript how and for what purpose the differential exon usage (DEU) analysis was performed. Differential gene expression (DEG) analysis allows the identification of genes with changes in overall expression levels between groups. However, this approach cannot detect changes occurring within a gene locus, such as alternative splicing events, alternative promoter usage, or differences in 3′ UTR usage. Therefore, we performed DEU analysis as a complementary approach to identify exon-level changes that may reflect post-transcriptional regulatory mechanisms. Such changes can alter protein domains or regulatory elements without necessarily affecting total gene expression levels (doi: 10.1101/gr.133744.111). To make this clearer, we made the following changes in the Results and Methods section:
Lines 368-369: "In addition to network propagation analysis, we investigated the post-transcriptional changes in exon usage of expressed genes by differential exon usage (DEU) analysis."
Lines 785-787: "Furthermore, to detect exon-level regulatory changes not captured by gene-level expression analyses, we performed DEU analysis using mapped RNA-seq reads with DEXSeq v.4.50.0, as described previously."
Lines 797-799: "The resulting set of candidate exons was subsequently overlapped with candidate variants to assess the potential effects of coding or noncoding variants on post-transcriptional regulation."

9. Where are the results of the HiC analysis? How were these data used? It is not clear. The relevant results are probably buried in the list of results.
Answer: We thank the reviewer for pointing out that the use of Hi-C data was not sufficiently clear. Hi-C data were used within the gABC framework to provide chromatin contact frequencies linking ATAC-seq-identified regulatory elements to their potential target genes. We have clarified this step in the Results section and added Hi-C sequencing statistics and all filtered output files for gABC-predicted regulatory element-gene interactions to a new Supplementary Data 6-file.
See Results section, line 284-287: Regulatory element-gene links were inferred using the generalised Activity-By-Contact (gABC) model, which integrates chromatin accessibility signals from ATAC-seq with chromatin contact frequencies derived from Hi-C (see Supplementary Data 6)."
See Methods section, Line 833-836: "Contact frequencies derived from Hi-C data together with chromatin accessibility signals from (read counts) from ATAC-seq peaks were used to predict regulatory element-target gene interactions. Interactions with a gABC score > 0.02 were retained."
**Supplementary Data 6**: Hi-C sequencing and mapping statistics from the mesenchymal stem cell sample obtained from one Mini-LEWE under study.

10. It would be useful to add a paragraph explaining why the authors decided to focus on these results (genes and lncRNA), as the information comes as somewhat of a surprise in the discussion. Maybe at the end of the Results section with Figure 3. It might be useful to have a scheme showing the relevant functional results.
Answer: We thank the reviewer for this helpful suggestion. The genes (including those encoding for lncRNAs) highlighted in the Discussion were prioritised based on the integration of multiple analyses performed in this study. To clarify this selection process for readers, we have added a short paragraph at the end of the Results section explaining the rationale for the prioritisation of candidate genes:
Line 373-376: "Finally, to prioritise biologically relevant candidates underlying miniature size, we integrated the results from the analyses described above. Candidate genes, including lncRNAs, supported by multiple lines of evidence, were considered the most likely functional candidates and are summarised in Fig. 4."
The integrated results are summarised in **Fig. 4**, which provides an overview of coding and non-coding candidate genes, DEGs, protein-protein interaction networks and enriched pathways for each miniature pig breed.

11. We would also be grateful if you could comment on the appropriateness and validity of any statistical analysis, as well the ability of a researcher to reproduce the work, given the level of detail provided.
Answer: We thank the reviewer for raising this important point regarding statistical rigor and reproducibility. The manuscript already provides detailed descriptions of all statistical analyses and computational procedures in the Methods section. Specifically, we report the software tools and versions used, parameter settings, normalization procedures, statistical models, thresholds applied for filtering, and the reference datasets employed throughout the analyses. These details allow the analyses to be reproduced as performed in this study.
In response to the reviewer's comment, we have carefully reviewed the Methods section again to ensure clarity and completeness. The statistical approaches applied in this study follow standard and widely accepted practices in genomics and multi-omics analyses.
We confirmed this in the manuscript section "Statistics and reproducibility".

12. The conclusion proposed by the authors is perhaps overly general.
We thank the reviewer for this comment. We have revised the concluding paragraph to avoid overly general statements and to better reflect the scope of our findings. In particular, clarified that our results contribute to understanding the gene-regulatory architecture associated with skeletal growth rather than implying direct functional mechanisms.
See conclusion, lines 525-531: "In summary, our data further support previous evidence pointing to the significant, though often underestimated, impact of noncoding variants in body size variation8,11. By integrating multiple omics layers in growth plate tissue of pigs, our study identified candidate regulatory variants and genes specifically under selection for miniature size. We propose that this dissection of the complex growth plate architecture into its

functional components offers new insights into the regulatory architecture underlying skeletal growth."

Minor comments

13. Line 56: Which lncRNA?

Amended.

14. Please avoid the abbreviation 'L2FC' in the legend of Figure 3b.

Amended.

15. According to supplementary data 2, there are 76 large and 32 small animals (1 NA), whereas lines 209–211 state that there are 65 large, 32 small, and 12 crossbred animals.

Answer: We thank the reviewer for this observation and have corrected the numbers accordingly.

Line 216-219: "Short-read whole-genome sequencing (WGS) data from four AM and six ML as well as an additional 22 miniature pigs (from six breeds/populations), 65 large pigs (from 18 breeds), one MinipigXMangalitza crossbreed and 11 larger-sized crossbred pigs from all over the world were analysed (Supplementary Data 2)."

16. Line 106: How many litters?

Answer: Amended, see line 107-109: We analysed genomic variants in ROHs as signatures of selection for miniature size in one litter of each of the two miniature pig breeds, Aachen Minipig (AM) and Mini-LEWE (ML),….

17. Line 305: Supplementary data 6, there is only 135 and not 136 lines. The results for SSC18 is missing.

Answer: We thank the reviewer for noticing this typo in the Suppl. Data (now Supplementary Data 7), which was corrected. In addition to our original FST study, we did a follow-up analysis extended to an expanded dataset including a total of 201 pigs with 23 additional larger-sized pigs and 70 additional miniature pigs. The following findings were made:

Line 345-353: "In a second step, the $F_{ST}$ analysis was extended to an expanded dataset comprising 201 pigs, including additional miniature pig populations and further larger-sized breeds for comparison, to evaluate the robustness of the observed differentiation signals in a broader population context. In this extended analysis, most of the differentiation peaks identified in the initial analysis were no longer detected above the significance threshold. However, the two peak regions with 349 $F_{ST}$ window hits on chromosomes three and four remained significant, with slightly shifted genomic positions (chromosome 3: 38,710,001-43,790,000 bp; chromosome 4: 75,630,001- 82,740,000 bp), and showed increased differentiation signals."

Line 861-864: "Furthermore, in order to evaluate the robustness of the observed differentiation signals in a broader population context, $F_{ST}$ analysis was extended to a panel of

201 pigs using additional data from Sequence Read Archive (SRA) including 23 larger-sized pigs and 70 miniature pigs mainly designated as Chinese local breeds."
Supplementary Data 7 has been updated accordingly.

18. Line 568: Please explain these numbers.

Answer: We corrected the sentence for more clarity:

Line 605-606: In total, we acquired 82 tissue sections from all four pigs (8-15 sections per location per breed), of which six images were blurry and removed from further analysis.

19. Line 588: Where does the classifier come from?

Answer: We thank the reviewer for pointing this out. The classifier used in this analysis is implemented in the ZEN 3.5 software. To improve clarity and readability, we now mention the software at the beginning of the paragraph rather than at the end (line 623-625).

20. Line 598: I assume this was done with R. Please specify.

Answer: We have addressed this by clarifying in the main text that the data was processed in R environment.

Lines 635-636: "In the next step, we used a linear mixed-effects model (lmer, lmerTest package v.3.1-3 and lme4 package v. 1.1-3374 in R environment)…"

21. Lines 687-705: Please give the number of samples obtained from how many animals for HiC. Same question for ATACseq (Lines 707-716).

Answer: Amended.

Hi-C, see lines 724-727: "We performed high-throughput chromatin conformation capture using the Hi-C technique to study genome-wide chromatin interactions within the nucleus of a bone-marrow-derived mesenchymal stem cell (MSC)-sample isolated from one of the studied ML piglets from the hip area."

ATAC-seq, see lines 744-746: "Open chromatin regions (OCRs) were identified using the Assay of Transposase Accessible Chromatin sequencing (ATAC-seq; n=24: 12 piglets sampled from humerus and femur each)…"

Further details on samples and sequencing method can be found in **Supplementary Data 2** and **Supplementary Data 12** (graphical summary).

22. Line 780: How many samples per analysis?

Answer: We thank the reviewer for this question. To improve clarity regarding the number of samples used in each analysis, we have added a **Supplementary Data 12**-file summarizing all animals included in the study and the corresponding sequencing datasets and analyses performed (e.g. WGS, RNA-seq, ATAC-seq, Hi-C, and subsequent analyses like gABC). This overview allows readers to easily identify the sample numbers for each analysis without interrupting the flow of the Methods section.

23. Line 830: Please clarify, three independent samples or three samples from the same humerus?

Answer: We thank the reviewer for pointing this out. We have clarified in the manuscript that these samples represent biological triplicates, derived from three different individuals.

Line 891: "Flash-frozen samples of growth plates from the left humerus, in biological triplicates, were immersed in cold lysis buffer…"

24. Lines 860-861: ConsensusPathDB-human is not only a PPI network but also "genetic, metabolic, signaling, gene regulatory and drug-target interactions, as well as biochemical pathways". This is important unless the authors have chosen a subset of information to construct the networks. Please clarify. The same for line 333.

Answer: We agree, the ConsensusPathDB contains interactions of many different types. However, in our analysis we focused only on the PPI part of the information to be consistent. We clarified this in the methods section.

See lines 924-928: "While the ConsensusPathDB contains different heterogeneous interactions, we focused on PPIs that were experimentally measured and evaluated with a quality score that is composed of annotation-based and topology-based measures to quantify the confidence associated with each given interaction (range [0,1])."

25. Line 880: How many nodes were kept at this stage? Is it the 2400?

Answer: We thank the reviewer for this question. This number 2400 refers to the nodes that had a pig homologue and for which proteomic and transcriptomic data were available from the experiments. Thus, it refers to the number of nodes that were initialized with a positive score (note that this differs slightly in the 4 comparisons: AMvsAS: 2372 proteins, AMvsMA: 2371, MLvsAS: 2372 and MLvsMA: 2370). All other nodes were weighted with zero. After the initialization step, however, network propagation was performed with the entire network in order to identify network modules that aggregate most of the weights associated with the observed differences between small and large pigs. The whole procedure was performed for each pairwise comparison (AMvsAS, AMvsMA, MLvsAS, and MLvsMA), resulting in four distinct sets of network modules.

26. Line 901-905: Please check what is declared. This is the method section.

Answer: We thank the reviewer for pointing this out. We agree that the previous wording in this section was too general. We have therefore revised the "Statistics and reproducibility" paragraph in the Methods section to more clearly describe the statistical approaches and the measures taken to ensure reproducibility.

27. Line 920: I wasn't able to access the PXD069390 dataset. It might be useful to provide a direct link to it.

Answer: We appreciate the reviewer's attention to this issue and apologize for the missing information. The dataset has been deposited and will be made publicly available upon publication. However, the submitted data can be accessed using the following login credentials:

Submission details:
PROJECT NAME: Integrative multi-omics analysis of growth plate regulation underlying body size in miniature pigs
REVIEWER ACCESS DETAILS Log in to the PRIDE website using the following details:
PROJECT ACCESSION: PXD069390
TOKEN: VdQH6eiifH0H Alternatively, the reviewer can access the dataset by logging in to the PRIDE website using the following account details:
USERNAME: reviewer_pxd069390@ebi.ac.uk
PASSWORD: Lkj86s8a1dpK

28. Supplementary data 7: What do the genotypes in columns AG to EK correspond to? Answer: We thank the reviewer for noting this issue. The genotype columns (AG–EK) in **Supplementary Data 8** (has been renamed due to a new Suppl. File) were inadvertently retained during file preparation and were not required for the analyses presented in the manuscript. These columns have now been removed, and the supplementary file has been updated accordingly.

Reviewer #3 (Remarks to the Author):
--------------------------------------
The authors have used an integrative approach, combining fine phenotyping, anatomical/histological studies and multi-omics analyses, to identify genes/variants regulating body size in miniature pigs. The topic is of interest to a wide scientific community. The authors have performed an impressive piece of work, generating loads of different data. Each analysis is well done and described.
However the current version of the manuscript is more like a catalogue of candidate genes and the authors don't discuss the potential molecular mechanisms or how those identified genetic variants might impact body size. In addition, no molecular functional validation is done. I thus found some of the conclusions quite speculative. For example, how the noncoding variant targeting the lncRNA ENSSSCG00000048200 impact body size?
I therefore recommend a major revision of the manuscript.
I've few questions:
1-I wonder why the ROH analyses were only done for the 2 miniature pig breeds and not also for the 2 large sized pig breeds. Human selection might have induced ROHR associated with body size in large sized pigs, as well.
Answer: We thank the reviewer for raising this important point. In this study, ROH analyses were primarily used to identify genomic regions that have become fixed or nearly fixed within the miniature pig breeds as a result of strong selection for reduced body size. Our aim was therefore to detect miniature-specific runs of homozygosity that may harbour variants contributing to the miniature phenotype. The large-sized pig breeds were included mainly as comparative populations representing larger body size rather than as populations expected to share the same selective pressure but for size increase. For this reason, the ROH analysis

focused on the miniature breeds, while the large-sized pigs were used as reference groups in downstream comparisons.

2- I worry that by focussing on variants/genes only found in ROHR identified in miniature pigs, the authors might be missing other variants associated with body size.
Answer: We agree that restricting the analysis to ROH regions in miniature pigs represents a prioritization strategy and may not capture all variants contributing to body size variation. Our rationale was to enrich for genomic regions that are fixed or nearly fixed in miniature breeds as a consequence of strong artificial selection and demographic bottlenecks, thereby increasing power to detect candidate variants underlying size reduction. Importantly, the ROH-based filtering was combined with multi-omics evidence (RNA-seq, ATAC-seq, and Hi-C), which allowed functional prioritization of non-coding variants with predicted regulatory effects in growth plate tissue.
In addition, the use of the Duroc-based reference genome (a large-sized breed) inherently increases sensitivity for detecting miniature-specific derived variants, because alleles fixed in miniature pigs are more likely to appear as non-reference variants. This asymmetry supports our focus on ROH regions in miniature populations.
We have now clarified in the manuscript that our approach is designed to identify high-confidence miniature-associated candidate loci rather than to provide an exhaustive catalog of all size-associated variants.
See discussion in lines 411-415: “To prioritise key growth-determining factors, our strategy combined breed selection with comparable growth patterns, a focus on ROHRs specific to miniature pigs expected to harbour variants fixed by strong artificial selection, and the selection of a developmental time point reflecting growth activity while minimising major sex-related hormonal influences.”

3- The authours didn't use the eQTL data from the FarmGTEx consortium. It'll be interesting to see if any of the identifed variants are also cis or trans eQTL SNPs.
Answer: We thank the reviewer for this helpful suggestion. The FarmGTEx consortium provides valuable eQTL resources for several porcine tissues; however, datasets from growth plate tissue, the primary tissue of interest in our study, are currently not available. Because eQTL effects are often highly tissue-specific, direct integration of FarmGTEx results would therefore be difficult to interpret in the context of growth plate biology.
Instead, to incorporate external genetic evidence related to body size, we interrogated the NHGRI-EBI GWAS Catalog using the search term “height” to identify loci previously associated with stature. These loci were considered during the prioritization of candidate genes identified through our integrative multi-omics analysis. Our approach therefore combines external association evidence with tissue-specific regulatory information derived from growth plate data (RNA-seq, ATAC-seq, Hi-C, and proteomics).

4- I don't see what brings the exon usage analysis. How does it help in identifying the

variants/genes associated with body size in miniature pigs? Could you explain please? Answer: We thank the reviewer for raising this important point. We have clarified in the revised manuscript how and for what purpose the differential exon usage (DEU) analysis was performed. Differential gene expression (DEG) analysis allows the identification of genes with changes in overall expression levels between groups. However, this approach cannot detect changes occurring within a gene locus, such as alternative splicing events, alternative promoter usage, or differences in 3′ UTR usage. Therefore, we performed DEU analysis as a complementary approach to identify exon-level changes that may reflect post-transcriptional regulatory mechanisms. Such changes can alter protein domains or regulatory elements without necessarily affecting total gene expression levels (doi: 10.1101/gr.133744.111). To make this clearer, we made the following changes in the Results and Methods section:
Line 368-369: "In addition to network propagation analysis, we investigated the post-transcriptional changes in exon usage of expressed genes by differential exon usage (DEU) analysis."
Line 785-787: "Furthermore, to detect exon-level regulatory changes not captured by gene-level expression analyses, we performed DEU analysis using mapped RNA-seq reads with DEXSeq v.4.50.0, as described previously."
Line 797-799: "The resulting set of candidate exons was subsequently overlapped with candidate variants to assess the potential effects of coding or noncoding variants on post-transcriptional regulation."

I've few comments:
1- Please had the rsID for the variants when possible.
Answer: We thank the reviewer for this suggestion. Where available, rsID identifiers from dbSNP have now been added for the reported variants in the manuscript and supplementary tables (see also Var Info in Suppl. Data 4).
See lines 330-332: "It was interesting to find that PLAG1 was also among the height-associated GWAS genes (GWAS catalogue) expressed in the growth plate, to which we could assign a homozygous alternative SNP (g.75646178:G>A, rs7605500220) in AM within ROHR."

2- Please rephrase L315: "homozygous mutant variant (g.75646178-75646178:G>A)" as "homozygous alternative SNP (rsID xx, g.75646178:G>A)"
Amended.

3- Be careful in the use of mutant instead of alternative allele.
Answer: We thank the reviewer for pointing this out. The term "mutant allele" has been replaced with "alternative allele" throughout the manuscript.

4- Please mentioned the variants (rsID and/or chromosome/position/alleles) when discussed (i.e. L418).
Amended. Line 452-453: "In AM, a noncoding variant (rs342683522) targeting an lncRNA encoded by ENSSSCG00000048200,…"

**Editors' response:**
If you decide to submit a revised version, we ask that your manuscript complies with our editorial policies. Specifically:
1. For all graphs depicting a single point value (e.g., mean) with error bars, you must add individual data points or convert the graph to a boxplot or dot-plot to show data distribution.
All figures were checked and already comply with these guidelines.

2. It's mandatory to provide access to the numerical source data for graphs and charts either through a repository or by providing the data in a Supplementary Data file (in excel format).
Sequencing data are available at SRA or PRIDE website upon publication. We made sure that it is fully accessible for the reviewers. Further data and statistics were provided in the Supplementary Data.

3. All blots/gels must be accompanied by size markers in every figure panel. Uncropped and unedited blot/gel images must be included as Supplementary Figure(s) in the Supplementary Information pdf.
Not applicable.

4. Please ensure that you have complied with the data deposition policies at the Nature Portfolio, please see here.
Checked.

5. Please ensure that you have complied with our policies on research involving animals and humans, see here
Checked.

6. Please follow the ARRIVE guidelines for reporting animal experiments. Please fully complete an ARRIVE checklist including both the essential and recommended set of items (adding information to the manuscript where needed) and upload this with your revised manuscript.
Provided.

7. Please also see our revision checklist for guidance on formatting the manuscript and complying with our policies. A comprehensive guide to our formatting requirements for final submissions is also available for your reference here.
Checked.
